# The methodological reporting quality in strictly randomized controlled trials for COVID-19 and precise reporting of Chinese herbal medicine formula intervention

**DOI:** 10.3389/fphar.2025.1532290

**Published:** 2025-03-31

**Authors:** Min-Li Chen, Shi-Yan Qian, Jiang-Li Yang, Jue-Yan Zheng, Li-Xiang Wang, Jing-Ying Wu, Hai-Qin Ye, Yan Wang, Guo-Qing Zheng

**Affiliations:** ^1^ Science and Technology Innovation Center, Guangzhou University of Chinese Medicine, Guangzhou, China; ^2^ Department of Neurology, The First Affiliated Hospital of Zhejiang Chinese Medical University (Zhejiang Provincial Hospital of Chinese Medicine), Hangzhou, China; ^3^ Institute of Developmental and Regenerative Biology, Zhejiang Key Laboratory of Organ Development and Regeneration, College of Life and Environmental Sciences, Hangzhou Normal University, Hangzhou, China; ^4^ Department of Cardiology, The First Affiliated Hospital of Zhejiang Chinese Medical University (Zhejiang Provincial Hospital of Chinese Medicine), Hangzhou, China

**Keywords:** Chinese herbal medicine, COVID-19, RCT, CONSORT-CHM formulas, subquestions, risk of bias

## Abstract

**Background:**

Chinese herbal medicine (CHM) formulas played an important role during the pandemic of coronavirus disease 2019 (COVID-19). Many randomized controlled trials (RCTs) on CHM for COVID-19 were quickly published. Concerns have been raised about their quality. In addition, inadequate detailed information on CHM formula intervention may arouse suspicion about their effectiveness. We aim to assess the most recent evidence of the methodological reporting quality of these RCTs with strict randomization, and the precise reporting of the CHM formula intervention.

**Methods:**

RCTs on CHM formulas for COVID-19 were searched from nine databases. The CONSORT 2010, CONSORT-CHM Formulas 2017, and risk of bias were the guidelines used to assess the included RCTs. The checklist of sub-questions based on CONSORT-CHM Formulas 2017 was used to evaluate the precise reporting of CHM formula intervention. A comparison was made between RCTs that enrolled participants during and after the first wave of the pandemic (defined here as December 2019 to March 2020).

**Results:**

The average score for 66 studies evaluated based on three guidelines, the CONSORT 2010, the CONSORT-CHM Formulas 2017, and the checklist of sub-questions based on the CONSORT-CHM Formulas 2017, is 16.4, 15.2, and 17.2, respectively. The reporting rate of sample size calculation, allocation concealment, and blinding is less than 30%. The checklist of sub-questions based on the CONSORT-CHM formulas 2017 can help report and assess CHM formula intervention more precisely. Most studies assessed an “unclear risk of bias” due to insufficient information. RCTs published in English and recruited subjects during the first wave of the pandemic have a higher risk of participant blinding bias than the studies recruited subjects after that (*P* < 0.05).

**Conclusion:**

The methodological reporting quality in strictly randomized RCTs on CHM formulas for COVID-19 is inadequate—the reporting of sample size calculation, allocation concealment, and blinding need to improve especially. The checklist of sub-questions based on CONSORT-CHM formulas 2017 can help report and assess CHM formula intervention more precisely. The methodological reporting quality of RCTs published in English and enrolled participants during the first wave of the pandemic is worse than the studies that recruited subjects after the first wave of the pandemic.

## 1 Introduction

The coronavirus disease 2019 (COVID-19) pandemic is one of the most serious challenges facing contemporary medicine. According to the World Health Organization, as of 21 July 2024, over 775 million confirmed cases and over 7 million deaths have been reported globally (WHO, 2024). The pandemic of COVID-19 has challenged scientific researchers to produce timely evidence about the new coronavirus and the disease. There has been a surge in the studies on COVID-19 since the pandemic started in 2020 (Ioannidis et al., 2021). In addition, the COVID-19 pandemic rapidly increases public interest concerning Chinese herbal medicine (CHM) (Rokhmah et al., 2020). One of the main issues that have been brought up is the caliber of the literature, considering synthesis studies are published quickly (Baumeister et al., 2021; Ang et al., 2022).

The best available data to assess the safety and curative effectiveness of therapies is typically found in well-conducted randomized controlled trials (RCTs), particularly in double-blind, placebo-controlled clinical studies. Evidence from well-designed RCTs is needed to conclusively identify what interventions should be applied or discontinued. Evaluating the methodological reporting quality of RCTs can provide more solid evidence for readers to judge the objectivity and reliability of the results of RCTs.

Clinical trial reports must be transparent, comprehensive, and easy to understand. It is imperative to evaluate the methodological reporting quality of RCTs with authoritative tools. One such tool for raising the methodological reporting standards of RCTs is the Consolidated Standards of Reporting Trials 2010 (CONSORT 2010) statement (Moher et al., 2012). Another is CONSORT-CHM Formulas 2017 (Cheng et al., 2017), which enhances the reporting quality of RCTs related to CHM formulas by adding traditional Chinese medicine (TCM) patterns and other items factors based on the features of CHM formulas. It is worth noting that the item of “intervention” of CONSORT-CHM Formulas 2017 contains many specific sub-items that well reflect detailed information on CHM formula interventions. However, there is hardly any literature that can be fully reported on this item because it doesn’t expand subitems for scoring. Some researchers created a 42 sub-questions checklist based on CONSORT-CHM formulas 2017 to improve the scoring process (Wang et al., 2024). To ensure uniformity throughout this assessment process, a standard operating procedure (SOP) for quality evaluation was developed. Furthermore, the risk of bias and the reporting quality of studies were always assessed using the Cochrane Collaboration’s Risk of Bias (RoB) tool (Higgins et al., 2022).

To date, several overviews assessing the quality of systematic reviews of COVID-19 with CHM treatment have been published (Jeona et al., 2022; Luo et al., 2022). However, only one research (Zhou et al., 2023a) on the quality appraisal of RCTs concerning COVID-19 treatment with traditional Chinese medicine (TCM). The researchers did a careful analysis and early quality evaluation of these RCTs. Nevertheless, there are some limitations: First of all, complete randomness is one of the core features of RCT. We found that some articles described themselves as RCTs but expressed random methods inaccurately, such as using only the word “random” without specifying the specific random method, or non-random methods. The research didn’t restrict the complete random methods of RCTs. Second, the research used only two evaluation tools, the CONSORT-CHM Formulas 2017 and RoB. Third, the research only searched literature from three databases, with a time from the establishment of the databases to 17 February 2023. Therefore, in our study, we aim to conduct a wider literature search to assess the most recent evidence of the methodological reporting quality of RCTs on CHM for COVID-19 with strict randomization, and the precise reporting of CHM formula intervention. Firstly, we searched nine databases and thoroughly searched literature between 1 January 2019, and 22 April 2024. We also analyzed the difference between the studies recruited subjects during and after the first wave of the pandemic (defined here as December 2019 to March 2020 inclusive because by this time the first wave of the pandemic had been largely contained within China) (Commission and Medicine, 2020a). Secondly, we include RCTs that use the random number table method, coin toss method, and other completely random methods, which are called strictly random RCTs here. In addition, inadequate detailed information regarding CHM formula interventions may lead to doubts about their effectiveness, so we use the checklist of 42 sub-questions based on CONSORT-CHM Formulas 2017 to evaluate the precise reporting of RCTs with CHM formula interventions.

## 2 Materials and methods

### 2.1 Information sources and search strategy

Nine electronic English and Chinese databases—the Cochrane Library, PubMed, Embase, Web of Science, EBSCO, China National Knowledge Infrastructure, VIP Journals Database, Wanfang Database, and Chinese Biomedical Database—were thoroughly searched between 1 January 2019, and 22 April 2024. The keywords used to search were as follows: “(COVID-19 OR SARS-CoV-2) AND (Traditional Chinese Medicine OR Chinese Herbal Drugs OR Integration of traditional Chinese and Western medicine) AND Randomized Controlled Trial”. The corresponding Chinese search terms were used for retrieval in the Chinese databases. The search results contained only publications in English and Chinese. To achieve an exhaustive search approach, free text words associated with the three basic topics were coupled with Medical Subject Headings (MeSH) terms and keywords. Supplementary Table 1 contains a comprehensive search strategy.

### 2.2 Article inclusion criteria

We included all RCTs evaluating CHM formulas as a standalone treatment or as an adjunct to standard care for COVID-19. At least one control group should receive no treatment, sham treatment, placebo, or routine care, regardless of publication status or language. The COVID-19 diagnosis was made in accordance with the diagnostic standards specified in the “COVID-19 Diagnosis and Treatment Protocol (Trial Sixth Version or later updated versions)” published by the National Administration of Traditional Medicine and the National Health Committee (Commission and Medicine, 2020b).

### 2.3 Exclusion criteria

The exclusion criteria encompassed non-clinical trials, observational experiments, reviews, meta-analyses, protocols, duplicate publications, trail registry records, abstracts, letters, communication, not strictly randomized RCTs (including trials with not randomized method such as using the odd-even grouping according to the last digit of the ID card, semi-random or no specific randomization method), non-COVID-19 confirmed patients, non-CHM formula-related studies and scientific and technological achievements registration form. Search is limited to English and Chinese.

### 2.4 Data extraction

Two investigators (Min-li Chen and Shi-yan Qian) underwent training to thoroughly examine every element and multiple sub-elements outlined in CONSORT 2010, CONSORT-CHM Formulas 2017, the checklist of sub-questions based on the CONSORT-CHM Formulas 2017, and risk of bias assessment to ensure a comprehensive understanding of each standard. Each report was evaluated by two independent researchers. They extracted data from the literature using the four evaluation tools mentioned above. To show whether the RCTs had reported the pertinent elements and sub-elements, the two authors separately awarded ratings of “1,” “0,” “NA,” or “NI.” “0” signifies partial disclosure or absence of the corresponding element/sub-element, while “1” indicates that the author had adequately described the element/sub-element. “NA” denotes “not applicable” and indicates that a specific element or sub-question is not relevant to a given study. “NI” signifies “no information” related to the element or sub-question. Supplementary Table 2 displays the precise data extraction as well as a comprehensive outline of the SOP of the checklist of sub-questions based on the CONSORT-CHM formula 2017. As for the risk of bias, the Cochrane Handbook for Systematic Reviews of Interventions’ definition of bias was used to guide the evaluation process. The items of risk of bias included sequence generation, allocation concealment, blinding of participants and personnel, blinding of outcome assessment, incomplete outcome data, selective outcome reporting, and other biases. The results were classified as “low risk of bias,” “unclear risk of bias,” or “high risk of bias” for each item. Supplementary Table 3 provides the evaluation criteria for the level of risk of bias. The specific assessment scores of the two researchers are shown in Supplementary Tables 4–7. If a score or judgment is inconsistent during the extraction process, the chief physician (Guo-qing Zheng) is called in to help resit the score.

### 2.5 Data analysis

For the descriptive statistical analysis, we used Microsoft Excel 2019, and we totaled the RCTs linked to each project. The results were expressed as percentages, and for each overall ratio, 95% confidence intervals (CIs) were calculated. SPSS (version 23.0) was used for statistical calculations, and a significance level of *p* < 0.05 was taken.

## 3 Results

### 3.1 Study selection

**FIGURE 1:**
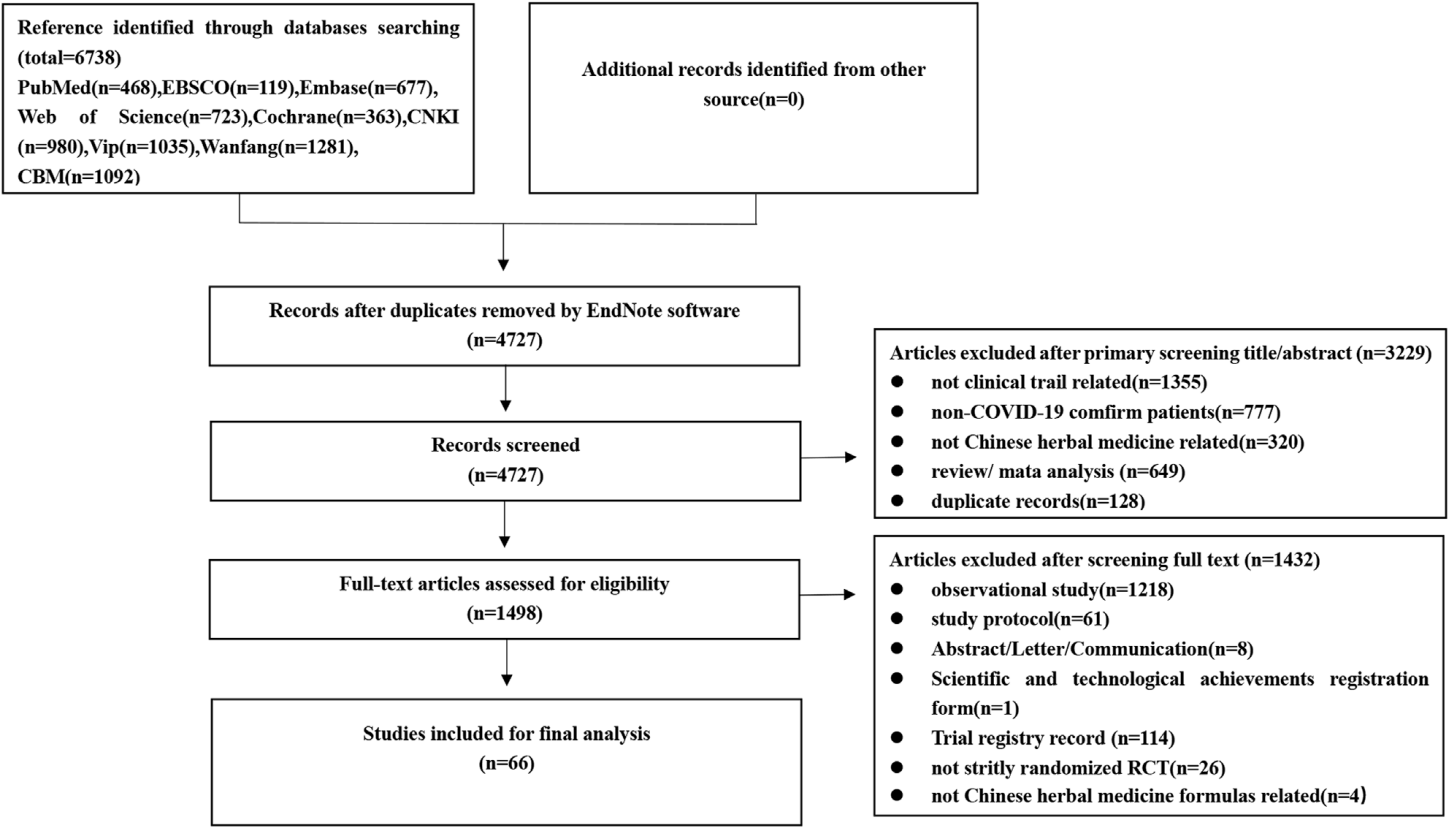
The flowchart of the study selection process.

A total of 6,738 articles that might be relevant were found. After using Endnote software to remove duplicates automatically, 4,727 papers remained. 3,229 papers were eliminated after titles and abstracts were reviewed, for one or more of the following reasons: 1) not a clinical trial, 2) review or meta-analysis, 3) duplicate publication, 4) non-COVID-19 confirmed patients, and 5) not related to Chinese herbal medicine. After further examination of the remaining literature by reading the full text, 1,432 papers were removed. Among these, 1,218 were observational studies, 61 were study protocols, 8 were abstracts or letters or communication, 1 were scientific and technological achievement registration form, 114 were trail registry records, 4 were not related to Chinese herbal medicine formulas, and 26 were not strictly randomized RCTs (Supplementary Tables 8, 9). Eventually, for the final analysis, 66 eligible RCT studies were chosen (Figure 1).

**FIGURE 2:**
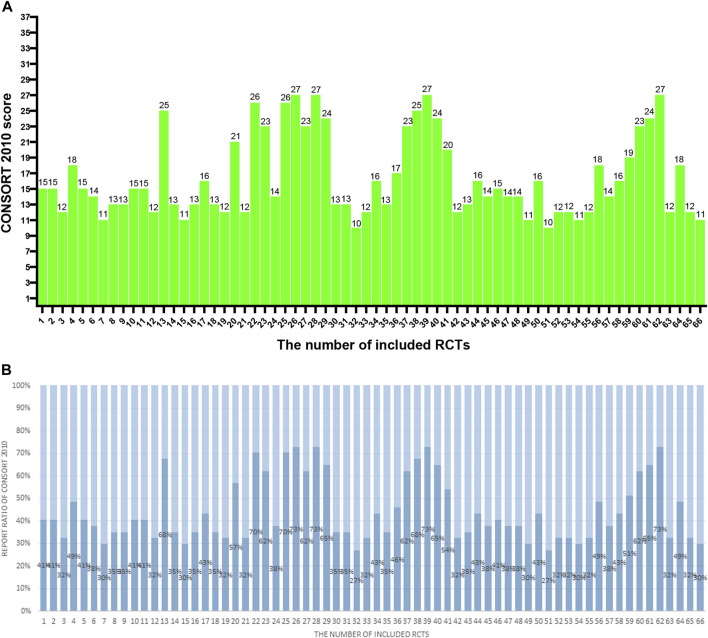
**(A)** The CONSORT 2010 scores of the 66 RCTs. The two researchers separately scored each of the included papers based on 37 items of the CONSORT 2010. If a score or judgment is inconsistent during the extraction process, the chief physician is called in to help resit the score. The figures represent the final scores of included RCTs. **(B)** The reported ratio of CONSORT 2010 of the 66 RCTs. The two researchers separately scored each of the included papers based on 37 items of the CONSORT 2010. If a score or judgment is inconsistent during the extraction process, the chief physician is called in to help resit the score. The figures represent the reported ratio of the CONSORT 2010 in included RCTs (score/37*100%).

**FIGURE 3:**
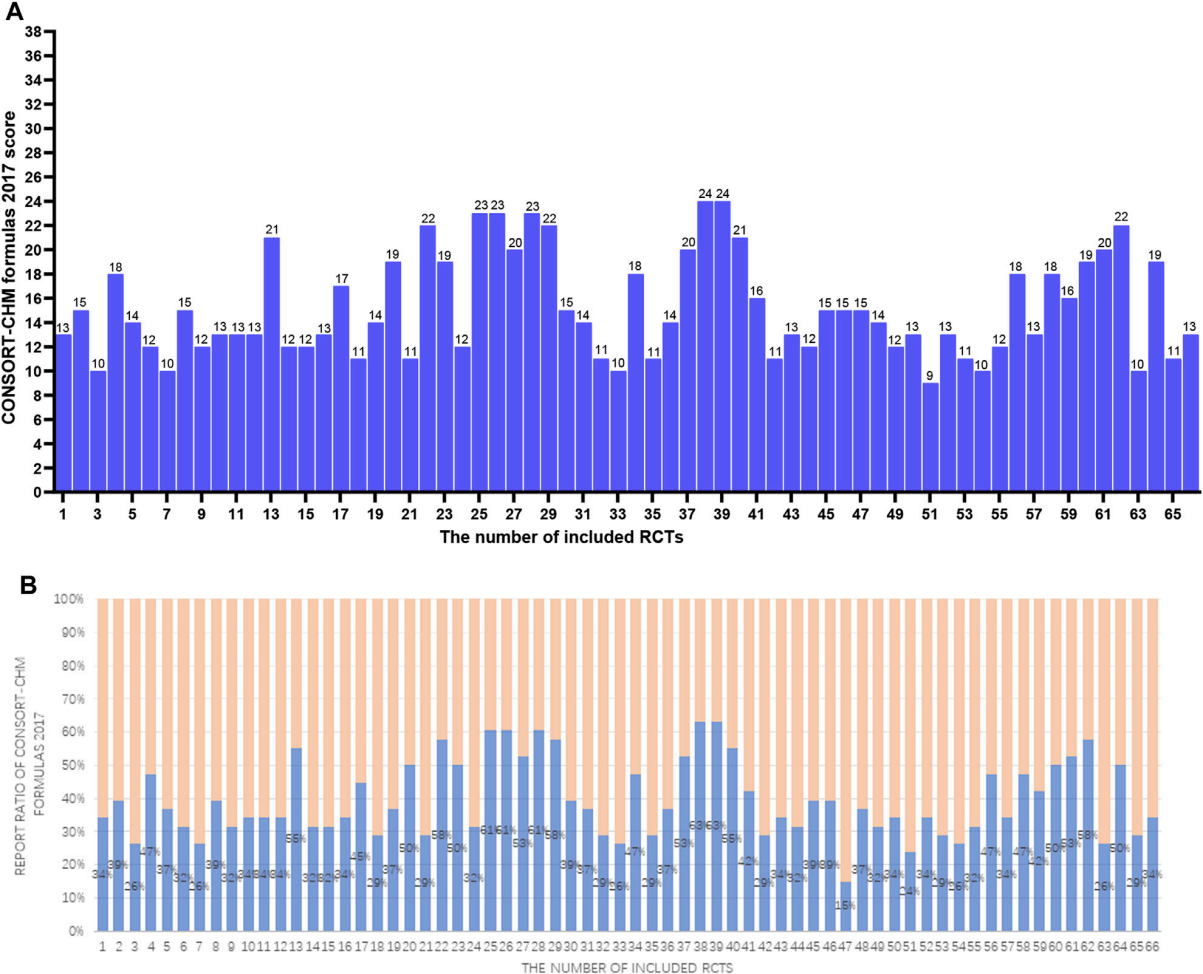
**(A)** The CONSORT-CHM formulas 2017 scores of the 66 RCTs. The two researchers separately scored each of the included papers based on 38 items of the CONSORT-CHM formulas 2017. If a score or judgment is inconsistent during the extraction process, the chief physician is called in to help resit the score. The figures represent the final scores of included RCTs. **(B)** The reported ratio of CONSORT-CHM Formulas 2017 of the 66 RCTs. The two researchers separately scored each of the included papers based on 38 items of the CONSORT-CHM formulas 2017. If a score or judgment is inconsistent during the extraction process, the chief physician is called in to help resit the score. The figures represent the reported ratio of the CONSORT-CHM Formulas 2017 in included RCTs (score/38*100%).

**FIGURE 4:**
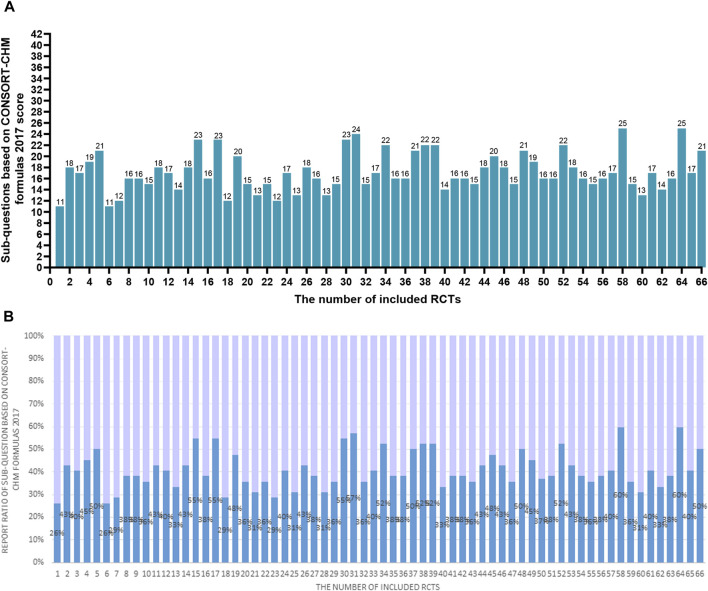
**(A)** The scores of the 66 RCTs evaluated based on the checklist of 42 sub-questions based on CONSORT-CHM Formulas 2017. The two researchers separately scored each of the included papers based on the checklist of 42 sub-questions based on the CONSORT-CHM formulas 2017. If a score or judgment is inconsistent during the extraction process, the chief physician is called in to help resit the score. The figures represent the final scores of included RCTs. **(B)** The reported ratio of the 66 RCTs evaluated based on the checklist of 42 sub-questions based on the CONSORT-CHM Formulas 2017. The two researchers separately scored each of the included papers based on the checklist of 42 sub-questions based on the CONSORT-CHM formulas 2017. If a score or judgment is inconsistent during the extraction process, the chief physician is called in to help resit the score. The figures represent the reported ratio of the checklist of 42 sub-questions based on the CONSORT-CHM Formulas 2017 in included RCTs (score/42*100%).

**FIGURE 5:**
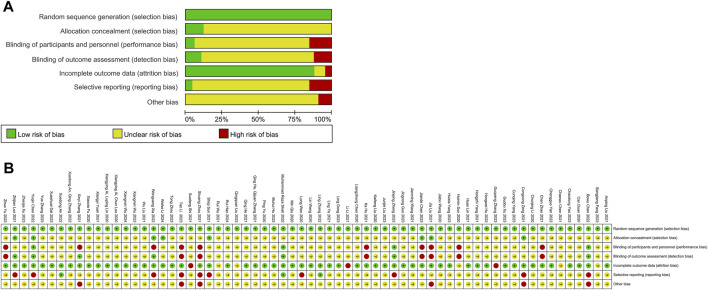
Risk of Bias. **(A)** Risk of bias of all included 66 RCTs; Risk of bias summary of all included 66 RCTs **(B)**. (Green indicates low risks, yellow indicates some concerns, and red indicates high risk).

**FIGURE 6:**
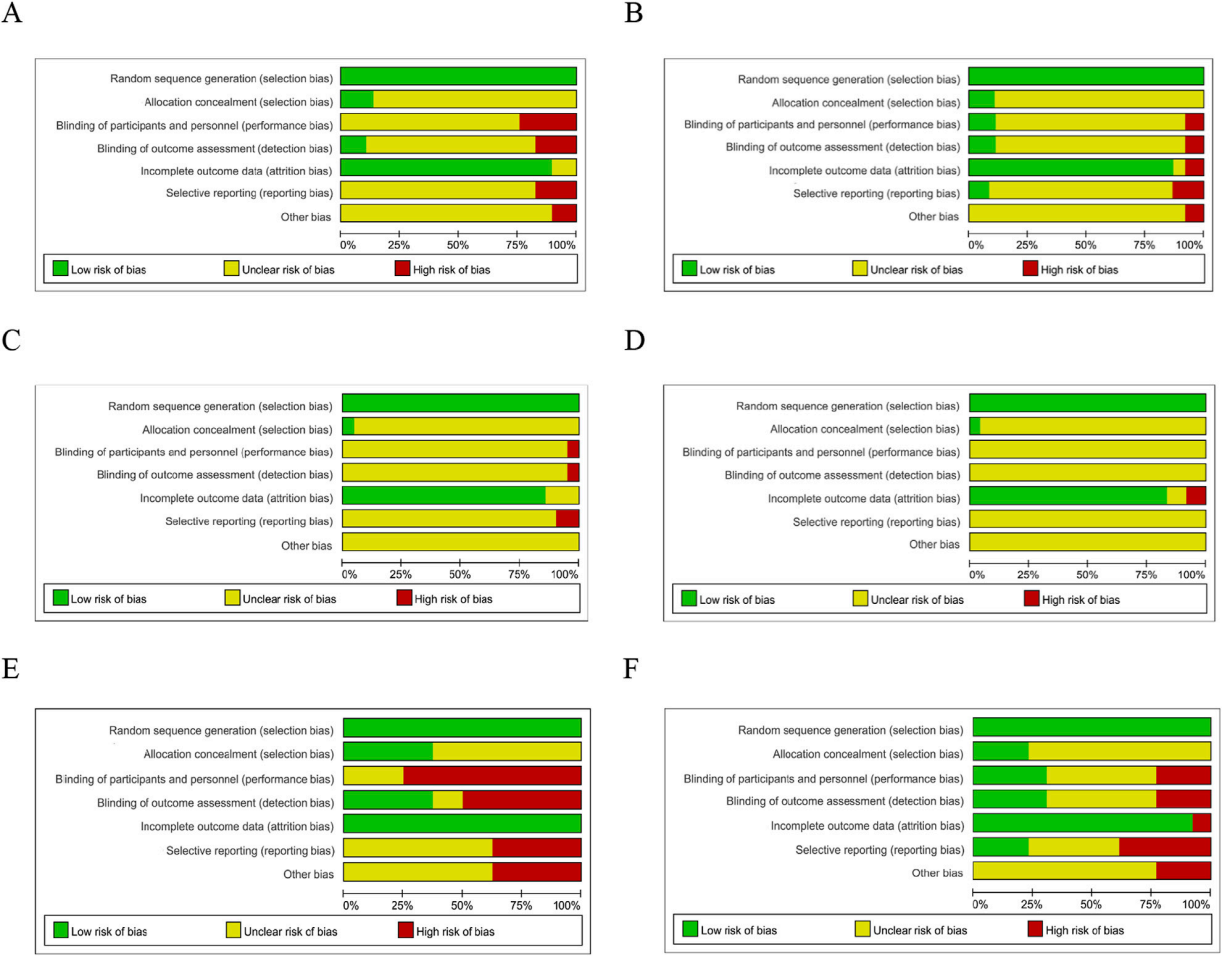
Risk of Bias. Risk of bias of RCTs recruited subjects during the first wave of the COVID-19 pandemic **(A)** and after the first wave of the COVID-19 pandemic **(B)**; Risk of bias of RCTs published in Chinese during the first wave of the COVID-19 pandemic **(C)** and after the first wave of the pandemic **(D)**; Risk of bias of RCTs published in English during the wave of the COVID-19 pandemic **(E)** and after the first wave of the pandemic **(F)**. (Green indicates low risks, yellow indicates some concerns, and red indicates high risk).

Sixty-six studies (Ai et al., 2020a; Ai et al., 2020b; An et al., 2022; An et al., 2023; Chen et al., 2022a; Chen et al., 2021; Chen et al., 2023; Chen et al., 2020; Chen et al., 2022b; Dai and Li, 2023; Dong et al., 2023; Duan et al., 2020; Fang et al., 2023; Fu et al., 2020; Guo et al., 2023; Han et al., 2024; He et al., 2021; He and Zhang, 2021; Hu et al., 2022; Hu et al., 2021; Hu, 2022; Li et al., 2020; Li et al., 2021; Li et al., 2023; Li et al., 2024; Lin et al., 2021; Lin et al., 2021; Liu et al., 2021a; Liu et al., 2021b; Liu, 2023; Liu et al., 2020; Liu et al., 2023; Liu et al., 2021c; Luo et al., 2020; Luo et al., 2021; Qiu et al., 2020; Shah et al., 2022; Shi et al., 2021; Sun et al., 2021a; Sun et al., 2021b; Wang et al., 2020; Wang et al., 2021; Wu et al., 2021; Wu et al., 2023; Wu et al., 2024; Xia et al., 2022; Xu et al., 2023a; Xu et al., 2023b; Yan et al., 2022; Yang et al., 2023; Yang, 2023; Yang et al., 2021; Yao, 2023; Ye et al., 2021; Yu et al., 2020; Yu et al., 2023; Yuan, 2021; Zeng et al., 2021; Zhang et al., 2023a; Zhang et al., 2022; Zhang et al., 2021; Zhang et al., 2023b; Zhao et al., 2021; Zheng et al., 2023; Zhou et al., 2021; Zhou et al., 2023b) involving 8712 COVID-19 patients were identified. For the 66 studies, there were 4,185 males and 4,360 females, and 4 studies did not mention the specific gender figures. Sample sizes ranged from 20 to 815 participants. The sample sizes of the included RCTs varied widely. Over time, there were more studies with larger sample sizes. This may be related to the increasing number of infections. Out of all 66 studies, 45 were published in Chinese and 21 in English. Sixty-four studies were conducted domestically, and two studies were conducted in collaboration between domestic and foreign investigators. More international cooperation is needed to carry out multi-center, large-sample RCTs to improve research representativeness and extrapolation. There were only thirty-three studies reported adverse effects. Adverse reactions are one of the important indicators to evaluate drug safety, and RCTs should report this part as much as possible. Five studies mentioned that they reported according to the CONSORT Statement. The CONSORT Statement standard has been published and used for many years. These self-reported RCTs that reported using this criterion did report a more complete picture of what was relevant to the study. Among other RCTs that do not report using this standard, some studies have good reporting quality, and there are more studies with poor reporting. Characteristics data are enumerated in Table 1; Supplementary Table 10. Table 2 summarizes the elements reported from the 66 RCTs in accordance with the CONSORT-CHM Formulas 2017 and the CONSORT 2010 statement. Table 3 summarizes the elements reported from the 66 RCTs based on sub-questions derived from the CONSORT-CHM Formulas 2017. Figures 2–4 display the distribution of the score and report ratio of the items on the three checklists of the included articles. The subgroup analysis results of RCTs recruited subjects during and after the first wave of the pandemic are shown in Table 4. The judgment of risk of bias is displayed in Figures 5, 6.

**TABLE 1:** Characteristics of included RCTs (n = 66).

Characteristics	Subgroups	n (%)
Types of journals	Chinese-language journal	45 (68.2%)
	English-language journal	21 (31.8%)
Publication year	2020	13 (19.7%)
	2021	21 (31.8%)
	2022	10 (15.2%)
	2023	19 (28.8)
	2024	3 (4.5%)
Participants	Adults	64 (97.0%)
	Children	2 (3.0%)
Type of Disease	Mild and/or moderate type	42 (63.6%)
	Severe and/or critical type	10 (15.1%)
	Convalescence	13 (19.7%)
	No report	6 (9.1%)
The country of recruitment	China	64 (97.0%)
	Pakistani	1 (1.5%)
	China and other countries	1 (1.5%)
The place of recruitment	Single center	51 (77.3%)
	Multicenter	15 (22.7%)
Baseline data	A table showing baseline demographic and clinical characteristics for each group	39 (59.1%)
Sample size	≤50	5 (7.6%)
	>50 and ≤100	33 (50%)
	>100	28 (42.4%)
Follow-up period	≤7 days	20 (30.3%)
	>7 and ≤14 days	35 (53.0%)
	>14 days	5 (7.6%)
	No report	6 (9.1%)
Controls	Traditional Chinese medicines	5 (7.6%)
	White placebo	9 (13.6%)
	Western medicines combined with basic treatment	51 (77.3%)
	No intervention	1 (1.5%)
Outcomes	Disappearance rate of clinical symptoms	29 (43.9%)
	Chinese medicine syndrome scores	28 (42.4%)
	Etiological outcomes	25 (37.8%)
	Imaging manifestations on the chest computed	18 (27.3%)
	Others	14 (21.2%)
Adverse events	Reported	33 (50%)
Ethics approved	Reported	44 (66.7%)
Informed consent	Reported	57 (86.4%)
Trial register	Reported	22 (33.3%)
Funding	Reported	35 (53%)
Report based on consort statement	Reported	5 (7.6%)

**TABLE 2:** The reporting number and percentage for each item of the CONSORT and CONSRT-CHM formulas checklist of the included 66 studies.

Section/Topic	Item no.	Checklist item	n	% (n/66)	95%CI
Title and abstract	1a	Identification as a randomized trial in the title	19	28.8	[18 to 40]
	1a*	Statement of whether the trial targets a TCM Pattern, a Western medicine–defined disease, or a Western medicine–defined disease with a specific TCM Pattern	66	100	[100 to 100]
	1b	Structured summary of trial design, methods, results, and conclusions (for specific guidance see CONSORT for abstracts)	66	100	[100 to 100]
	1b*	Illustration of the name and form of the formula used, and the TCM Pattern applied, if applicable	11	16.7	[8 to 26]
	1c*	Determination of appropriate keywords, including “Chinese herbal medicine formula” and “RCT”	6	9.1	[2 to 16]
Introduction					
Background and objectives	2a	Scientific background and explanation of the rationale	66	100	[100 to 100]
	2a*	Statement with biomedical science approaches and/or TCM approaches	64	97	[93 to 100]
	2b	Specific objectives or hypotheses	66	100	[100 to 100]
	2b*	Statement of whether the formula targets a Western medicine–defined disease, a TCM Pattern, or a Western medicine-defined disease with a specific TCM Pattern	66	100	[100 to 100]
Methods					
Trial design	3a	Description of trial design (such as parallel, factorial) including allocation ratio	15	22.7	[13 to 33]
	3b	Important changes to methods after trial commencement (such as eligibility criteria), with reasons	1	1.5	[-1 to 5]
Participants	4a	Eligibility criteria for participants	66	100	[100 to 100]
	4a*	Statement of whether participants with a specific TCM Pattern were recruited, in terms of 1) diagnostic criteria and 2) inclusion and exclusion criteria. All criteria used should be universally recognized, or reference given to where detailed explanations can be found	23	34.8	[23 to 46]
	4b	Settings and locations where the data were collected	58	87.9	[80 to 96]
Interventions	5	The interventions for each group with sufficient details to allow replication, including how and when they were actually administered	66	100	[100 to 100]
	5*	Description(s) for different types of formulas should include specific contents	0	0	[0 to 0]
Outcomes	6a	Completely defined prespecified primary and secondary outcome measures, including how and when they were assessed	21	31.8	[21 to 43]
	6a*	Illustration of outcome measures with Pattern in detail	26	39.4	[28 to 51]
	6b	Any changes to trial outcomes after the trial commenced, with reasons	1	1.5	[−1 to 5]
Sample size	7a	How the sample size was determined	14	21.2	[11 to 31]
	7b	When applicable, explanation of any interim analyses and stopping guidelines	1	1.5	[−1 to 5]
Randomization					
Sequence generation	8a	The method used to generate the random allocation sequence	66	100	[100 to 100]
	8b	Type of randomization; details of any restriction (such as blocking and block size)	7	10.6	[3 to 18]
Allocation concealment mechanism	9	The mechanism used to implement the random allocation sequence (such as sequentially numbered containers), describing any steps taken to conceal the sequence until interventions were assigned	10	15.2	[7 to 24]
Implementation	10	Who generated the random allocation sequence, who enrolled participants, and who assigned participants to interventions	12	18.2	[9 to 28]
Blinding	11a	If done, who was blinded after assignment to interventions (for example, participants, care providers, those assessing outcomes) and how	7	10.6	[3 to 18]
	11b	If relevant, a description of the similarity of interventions	5	7.6	[1 to 14]
Statistical methods	12a	Statistical methods used to compare groups for primary and secondary outcomes	66	100	[100 to 100]
	12b	Methods for additional analyses, such as subgroup analyses and adjusted analyses	4	6.1	[0 to 12]
Results					
Participant flow (a diagram is strongly recommended)	13a	For each group, the numbers of participants who were randomly assigned, who received intended treatment, and who were analyzed for the primary outcome	25	37.9	[26 to 50]
	13b	For each group, losses and exclusions after randomization, together with reasons	30	45.5	[33 to 58]
Recruitment	14a	Dates defining the periods of recruitment and follow-up	33	50	[38 to 62]
	14b	Why the trial ended or was stopped	1	1.5	[−1 to 5]
Baseline data	15	A table showing baseline demographic and clinical characteristics for each group	39	59.1	[47 to 71]
Baseline data	16	For each group, the number of participants (denominator)included in each analysis and whether the analysis was by the originally assigned groups	59	89.4	[82 to 97]
Outcomes and estimation	17a	For each primary and secondary outcome, results for each group, and the estimated effect size and its precision (such as 95% confidence interval)	6	9.1	[2 to 16]
	17b	For binary outcomes, the presentation of both absolute and relative effect sizes is recommended	66	100	[100 to 100]
Ancillary analyses	18	Results of any other analyses performed, including subgroup analyses and adjusted analyses, distinguishing prespecified from exploratory	4	6.1	[0 to 12]
Harms	19	All important harms or unintended effects in each group (for specific guidance see CONSORT for harms)	55	83.3	[74 to 92]
Discussion					
Limitations	20	Trial limitations, addressing sources of potential bias, imprecision, and, if relevant, multiplicity of analyses	39	59.1	[47 to 71]
Generalizability	21	Generalizability (external validity, applicability) of the trial findings	1	1.5	[−1 to 5]
	21*	Discussion of how the formula works on different TCM Patterns or diseases	1	1.5	[−1 to 5]
Interpretation	22	Interpretation consistent with results, balancing benefits and harms, and considering other relevant evidence	19	28.8	[18 to 40]
	22*	Interpretation with TCM theory	49	74.2	[64 to 85]
Other information					
Registration	23	Registration number and name of trial registry	21	31.8	[21 to 43]
Protocol	24	Where the full trial protocol can be accessed, if available	6	9.1	[2 to 16]
Funding	25	Sources of funding and other support (such as a supply of drugs), role of funders	36	54.5	[43 to 67]
The total mean score of CONSORT a			16.4 ± 5.2		
The total mean score of CONSORT-CHM a			15.2 ± 4.2		

CONSORT-CHM = CONSORT, an extension for Chinese herbal medicine formulas. Items with * indicate that this item is CONSORT-CHM a indicates Mean ± SD.

**TABLE 3:** The checklist of 42 Sub-questions based on the CONSORT-CHM Formula 2017.

Section/topic	Extension items	Sub-questions for assessment	n	% (n/66)	95%CI
Title, abstract, and keywords	1a. Statement of whether the trial targets a TCM Pattern, a Western medicine–defined disease, or a Western medicine–defined disease with a specific TCM Pattern, if applicable	Q1. Whether it reported that the trial targeted a specific TCM Pattern in “Title”?	9	13.6	[5 to 22]
	1b. Illustration of the name and form of the formula used, and the TCM Pattern applied, if applicable	Q2. Whether the name of the CHM formula was reported in the “Abstract”?	63	95.5	[90 to 100]
		Q3. Whether the dosage form of the CHM formula reported in the “Abstract”?	64	97	[93 to 100]
		Q4. Whether the TCM Pattern was reported in “Abstract”?	11	16.7	[8 to 26]
	1c. Determination of appropriate keywords, including “Chinese herbal medicine formula” and “randomized controlled trial”	Q5. Whether the “Chinese herbal medicine formula” presented in “Keyword”?	54	81.8	[73 to 91]
		Q6. Whether “randomized controlled trials” was presented in “Keywords”?	7	10.6	[3 to 18]
Introduction	2a. Statement with biomedical science approaches and/or TCM approaches	Q7. Whether the TCM background and explanation of the disease or the TCM Pattern was reported in “Background”?	16	24.2	[14 to 35]
Background and objectives		Q8. Whether the biomedical science explanation and/or TCM rationale about the CHM formula were reported in “Background”?	66	100	[100 to 100]
	2b. Statement of whether the formula targets a Western medicine–defined disease, a TCM Pattern, or a Western medicine–defined disease with a specific TCM Pattern	Q9. Whether the objective or hypotheses focused on the CHM formula in the treatment of a Western medicine-defined disease, a TCM Pattern, or a Western medicine-defined disease with a specific TCM Pattern?	10	15.2	[7 to 24]
Methods	4a.Statement of whether participants with specific TCM Patterns were recruited, in terms of 1) diagnostic criteria and 2) inclusion and exclusion criteria. All criteria used should be universally recognized, or reference is given to where a detailed explanation can be found	Q10. Whether the participants with a specific TCM Pattern recruited, in terms of 1) diagnostic criteria and 2) inclusion and exclusion criteria, and whether all criteria used universally recognized, or reference given to where a detailed explanation can be found in “Methods”?	24	36.4	[25 to 48]
Participants					
Interventions	5a-1. Name, source, and dosage form (e.g., decoctions, granules, powders)	Q11. Whether the name of the CHM formula was reported in “Methods”?	65	98.5	[96 to 100]
5a. For fixed CHM formulas		Q12. Whether the source of the CHM formula was reported in “methods”?	55	83.3	[74 to 92]
		Q13. Whether the dosage form of the CHM formula was reported in “methods”?	66	100	[100 to 100]
	5a-2. Name, source, processing method, and dosage of each medical substance. Names of substances should be presented in at least 2 languages: Chinese (Pinyin), Latin, or English. Names of the parts of the substances used should be specified	Q14. Whether the name of each medical substance was reported in “Methods”?	36	54.5	[43 to 67]
		Q15. Whether the source of each medical substance was reported in “Methods”?	19	28.8	[18 to 40]
		Q16. Whether the processing method of each medical substance was reported in “Methods”?	19	28.8	[18 to 40]
		Q17. Whether the dosage of each medical substance was reported in “Methods”?	27	40.9	[29 to 53]
	5a-3.Authentication method of each ingredient and how, when, where, and by whom it was conducted; statement of whether any voucher the specimen was retained, and if so, where they were kept and whether they are accessible	Q18.Whether the Authentication method of each ingredient reported in “Methods”?	0	0	[0 to 0]
	5a-4. Principles, rationale, and interpretation of forming the formula	Q19. Whether the principles, rationale, and interpretation of forming the formula were reported?	23	34.8	[23 to 46]
	5a-5. Reference(s) as to the efficacy of the formula, if any	Q20. Whether the reference(s) as to the efficacy of the formula was presented?	37	56.1	[44 to 68]
	5a-6. Pharmacologic study results of the formula, if any	Q21. Whether the pharmacologic study results of the formula presented?	38	57.6	[46 to 70]
	5a-7. The production method of the formula, if any	Q22. Whether the production method of the formula was reported?	22	33.3	[22 to 45]
	5a-8. Quality control of each ingredient and of the product of the formula, if any. This would include any quantitative and/or qualitative testing method(s); when, where, how, and by whom these tests were conducted; whether the original data and samples were kept, and, if so whether they are accessible	Q23. Whether the quality control of each ingredient and of the product of the formula was conducted?	2	3	[−1 to 7]
	5a-9. Safety assessment of the formula, including tests for heavy metals and toxic elements, pesticide residues, microbial limit, and acute/chronic toxicity, if any. If yes, it should be stated when, where, how, and by whom these tests were conducted; if the original data and samples were kept; and, if so, whether they are accessible	Q24. Whether the safety assessment of the formula was conducted?	0	0	[0 to 0]
	5a-10. Dosage of the formula, and how the dosage was determined	Q25. Whether the dosage of the formula reported?	44	66.7	[55 to 78]
		Q26. Whether the treatment duration of the CHM formulas was reported in “Methods”?	63	95.5	[90 to 100]
	5a-11. Administration route (e.g., oral, external)	Q27. Whether the Administration route of the CHM formula was reported in “Methods”?	66	100	[100 to 100]
5b. For individualized CHM formulas	5b-1. See recommendations 5a 1–11	See Q11 to Q27			
	5b-2. Additional information: how, when, and by whom the formula was modified	Q28. For trials with individualized CHM formulas, whether it reported how, when, and by whom the CHM formula was modified in “Methods”?	9	13.6	[5 to 22]
5c. For patent proprietary CHM formulas	5c-1. Reference to publicly available materials, such as pharmacopeia, for the details about the composition, dosage, efficacy, safety, and quality control of the formula	Q29. For trials with patent proprietary CHM formulas, whether the composition and dosage reported in “Methods”?	12	18.2	[9 to 28]
	5c-2. Illustration of the details of the formula, namely, 1) the proprietary product name (i.e., brand name),2) the name of the manufacturer, 3) lot number, 4) production date and expiry date, 5) name and percentage of added materials, and 6) whether any additional quality control measures were conducted	Q30. For trials with patent proprietary CHM formulas, whether the efficacy was reported in “Methods”?	26	39.4	[28 to 51]
		Q31. For trials with patent proprietary CHM formulas, whether the safety or quality control reported in “Methods”?	3	4.5	[-1 to 10]
		Q32. For trials with patent proprietary CHM formulas, whether the proprietary product name (i.e., brand name), name of the manufacturer, and lot number were reported in “Methods”?	22	33.3	[22 to 45]
		Q33. For trials with patent proprietary CHM formulas, whether the production date and expiry date were reported in “Methods”?	22	33.3	[22 to 45]
	5c-3. Statement of whether the patent proprietary formula used in the trial is for a condition that is identical to the publicly available reference	Q34. For trials with patent-proprietary CHM formulas, whether the patent-proprietary formula used in the trial for a condition that is identical to the publicly available reference stated?	28	42.4	[31 to 54]
5d. Control groups Placebo control	5d-1. Name and amount of each ingredient	Q35. For trials with placebo control, whether the name and amount of each ingredient of the placebo were reported in “Methods”?	0	0	[0 to 0]
	5d-2. Description of the similarity of placebo with the intervention (e.g., color, smell, taste, appearance, packaging)	Q36. For trials with placebo control, whether the similarity of placebo with the intervention (e.g., color, smell, taste, appearance, packaging) reported in “Methods”?	2	3	[−1 to 7]
	5d-3. Quality control and safety assessment, if any	Q37. For trials with placebo control, whether the quality control and safety assessment of the placebo were reported in “Methods”?	2	3	[−1 to 7]
	5d-4. Administration route, regimen, and dosage	Q38. For trials with placebo control, whether the administration route, regimen, and dosage of the placebo were reported in “Methods”?	9	13.6	[5 to 22]
	5d-5. Production information: where, when, how, and by whom the placebo was produced	Q39. For trials with placebo control, whether the production information of the placebo was reported, including where, when, how, and by whom the placebo was produced?	0	0	[0 to 0]
Outcomes	Illustration of outcome measures with Pattern in detail	Q40. Whether the outcome measures include TCM indicators in “Outcome”?	32	48.5	[36 to 61]
Discussion	Discussion of how the formula works on different TCM Patterns or diseases	Q41. Whether any discussion of how the formula works on different TCM Patterns or diseases reported in “Discussion”?	0	0	[0 to 0]
Generalizability					
Interpretation	Interpretation of TCM theory	Q42. Whether any interpretation with TCM theory reported in the “Discussion”?	50	75.8	[65 to 86]
The total mean score of a			17.2 ± 3.4		

TCM, traditional Chinese medicine; CHM, Chinese herbal medicine. a indicates Mean ± SD.

**TABLE 4:** The difference between RCTs recruited subjects during and after the first wave of the pandemic.

Item	Rob item	RCTs recruited subjects in the first wave of the pandemic (n = 29)	RCTs recruited subjects after the first wave of the pandemic (n = 37)	χ^2^ test χ^2^; *P*	RCTs published in Chinese and recruited subjects in the first wave of the pandemic. (n = 21)	RCTs published in Chinese and recruited subjects after the first wave of the pandemic (n = 24)	χ^2^ test χ^2^;*P*	RCTs published in English and recruited subjects in the first wave of the pandemic. (n = 8)	RCTs published in English and recruited subjects after the first wave of the pandemic (n = 13)	Fisher’s exact test *P* or χ^2^ test χ^2^; *P*
Low risk of bias	1. Sequence generation	29 (100%)	37 (100%)	—	21 (100%)	24 (100%)	—	8 (100%)	13 (100%)	—
	2. Allocation concealment	4 (14%)	4 (11%)	0; 1	1 (5%)	1 (4%)	0; 1	3 (38%)	3 (23%)	0.631
	3. Blinding of participants and personnel	0 (0%)	4 (11%)	1.709; 0.191	0 (0%)	0 (0%)	—	0 (0%)	4 (31%)	0.131
	4. Blinding of outcome assessment	3 (10%)	4 (11%)	0; 1	0 (0%)	0 (0%)	—	3 (38%)	4 (31%)	1
	5. Incomplete outcome data	27 (93%)	32 (86%)	0.215; 0.643	19 (90%)	20 (83%)	0.07; 0.792	8 (100%)	12 (92%)	0.131
	6. Selective outcome reporting	0 (0%)	3 (8%)	0.949; 0.330	0 (0%)	0 (0%)	—	0 (0%)	3 (23%)	0.257
	7. Other bias	0 (0%)	0 (0%)	—	0 (0%)	0 (0%)	—	0 (0%)	0 (0%)	—
High risk of bias	1. Sequence generation	0 (0%)	0 (0%)	—	0 (0%)	0 (0%)	—	0 (0%)	0 (0%)	—
	2. Allocation concealment	0 (0%)	0 (0%)	—	0 (0%)	0 (0%)	—	0 (0%)	0 (0%)	—
	3. Blinding of participants and personnel	7 (24%)	3 (8%)	2.122; 0.145	1 (5%)	0 (0%)	0.005; 0.946	6 (75%)	3 (23%)	0.032*
	4. Blinding of outcome assessment	5 (17%)	3 (8%)	0.560; 0.454	1 (5%)	0 (%)	0.005; 0.946	4 (50%)	3 (23%)	0.346
	5. Incomplete outcome data	0 (0%)	3 (8%)	0.949; 0.330	0 (0%)	2 (8%)	0.395; 0.53	0 (0%)	1 (8%)	1
	6. Selective outcome reporting	5 (17%)	5 (14%)	0.005; 0.942	2 (10%)	0 (%)	0.675; 0.411	3 (38%)	5 (38%)	1
	7. Other bias	3 (10%)	3 (8%)	0; 1	0 (0%)	0 (0%)	—	3 (38%)	3 (23%)	0.631
Unclear risk of bias	1. Sequence generation	0 (0%)	0 (0%)	—	0 (0%)	0 (0%)	—	0 (0%)	0 (0%)	—
	2. Allocation concealment	25 (86%)	33 (89%)	0; 1	20 (95%)	23 (96%)	0; 1	5 (63%)	10 (77%)	0.631
	3. Blinding of participants and personnel	22 (76%)	30 (81%)	0.045; 0.833	20 (95%)	24 (100%)	0.005; 0.946	2 (25%)	6 (46%)	0.4
	4. Blinding of outcome assessment	21 (72%)	30 (81%)	0.289; 0.591	20 (95%)	24 (100%)	0.005; 0.946	1 (13%)	6 (46%)	0.174
	5. Incomplete outcome data	2 (7%)	2 (5%)	0; 1	2 (10%)	2 (8%)	0; 1	0 (0%)	0 (0%)	—
	6. Selective outcome reporting	24 (83%)	29 (78%)	0.017; 0.895	19 (90%)	24 (100%)	0.675; 0.411	5 (63%)	5 (38%)	0.387
	7. Other bias	26 (90%)	34 (92%)	0; 1	21 (100%)	24 (100%)	—	5 (63%)	10 (77%)	0.6318
CONSORT (mean value)		17 (46%)	16 (43%)	0; 1	14 (38%)	13 (35%)	0.58; 0.809	25 (68%)	21 (57%)	0.919; 0.338
CONSORT (report 50%)		9 (31%)	9 (24%)	0.369; 0.544	1 (%)	0 (0%)	0.005; 0.946	8 (100%)	9 (69%)	0.131
CONSORT-CHM (mean value)		16 (42%)	15 (39%)	0.54; 0.815	13 (34%)	14 (37%)	0.057; 0.811	22 (58%)	18 (47%)	0.844; 0.358
CONSORT-CHM (report 50%)		9 (31%)	8 (22%)	0.753; 0.385	1 (5%)	1 (4%)	0; 1	8 (100%)	7 (54%)	0.046*
Sub-questions based on CONSORT-CHM(mean value)		16 (38%)	18 (43%)	0.198; 0.657	17 (40%)	19 (45%)	0.194; 0.659	14 (33%)	17 (40%)	0.46; 0.498
Sub-questions based on CONSORT-CHM(report 50%)		4 (14%)	10 (27%)	1.704; 0.192	4 (19%)	7 (29%)	0; 1	0 (0%)	3 (23%)	0.257
Trial register		10 (34%)	12 (32%)	0.031; 0.861	2 (10%)	0 (0%)	0.675; 0.411	8 (100%)	12 (92%)	1
Funding		18 (62%)	18 (49%)	1.181; 0.227	12 (57%)	11 (46%)	0.573; 0.449	6 (75%)	7 (54%)	0.4
Ethics approved		15 (52%)	29 (78%)	5.198; 0.023*	9 (43%)	17 (71%)	3.593; 0.058	6 (75%)	12 (92%)	0.531
Informed consent		24 (83%)	33 (89%)	0.155; 0.693	16 (76%)	20 (83%)	0.05; 0.823	8 (100%)	13 (100%)	—

P means the difference between RCTs, in and after the first wave of the pandemic. *means *P* < 0.05.

### 3.2 Quality of study

#### 3.2.1 CONSORT 2010

The results are shown in Table 2; Figure 2. Total mean scores Among the 66 articles in this study, the average score of the CONSORT 2010 evaluation is 16.4, accounting for only 44% of the total 37 items. The specific breakdown of 37 items is as follows:

##### 3.2.1.1 Title and abstract

After reviewing the title (1a), only 19 (28.8%) trials were found to meet the criteria for being classified as RCTs. Additionally, all of the articles (100%) included abstracts that provided a structured overview of the experimental design, methods, results, and conclusions (1b).

##### 3.2.1.2 Introduction

The total included studies mentioned a scientific background and explanation of the rationale (2a) and presented specific objectives or hypotheses (2b).

##### 3.2.1.3 Methods

All the articles included described two CONSORT 2010 items, with 100% coverage, which included a trial design with eligibility criteria for participants (4a) and detailed interventions for each group to enable replication (5). Settings and locations where the data were collected (4b) were described in 87.9% of the research. Of the publications, 22.7% revealed the trial design including participant allocation ratio (3a), and 31.8% described the primary and secondary outcome measures (6a). Fourteen articles (21.2%) explained how sample size was determined (7a). Sample size calculation is an important part of the RCT design process, but the report rate of sample size calculation in the included literature is not satisfactory. Only 1 (1.5%) study, respectively, described significant changes in the experimental method (3b), whether there were changes in the trial outcomes after the commencement of the experiment (6b), and information on the interim analysis and stopping guidelines (7b).

##### 3.2.1.4 Randomization

The process for creating the random allocation sequence (8a) as well as comprehensive statistical methods (12a) were offered in all sixty-six (100%) studies. However, only 10.6% of papers explained the kind of randomization (8b), ten papers (15.2%) reported using the random allocation sequence (9), 18.2% gave thorough information about the implementation (10), and 10.6% discussed the blinding procedure (11a), five studies (7.6%) mentioned intervention similarities (11b). The rate of reporting information about the implementation of the random allocation is low, and so is the rate of reporting on the blind method. Furthermore, 6.1% of publications included techniques for further analysis (12b).

##### 3.2.1.5 Results

Thirty-five (45.5%) studies provided an explanation of the losses and exclusions (13b) following randomization, while twenty-five (37.9%) studies used a diagram to show the treatment progress (13a). The treatment progress flow chart provides a clearer picture of the inclusion and loss of study subjects and is also relevant to the assessment of bias risk for reporting results fully. However, the reporting rate in this segment is less than 50%. Thirty-three studies (50%) provided the dates that defined the recruitment and follow-up periods (14a). Out of all the articles, just one (1.5%) explained why a trial was terminated or discontinued (14b). A total of thirty-nine (59.1%) articles included baseline information, which included basic demographic and clinical features as well as underlying disease (15). Baseline characteristics are expected to be reported especially. Because it plays an important role in evaluating the comparability between groups, improving the credibility of results, enhancing the extrapolation of results, statistical analysis, and so on. Fifty-nine (89.4%) studies described the number of participants of each group and whether the analysis was by originally assigned groups (16). The total of papers (100%) reported absolute or relative effect sizes (17b), while only six articles (9.1%) supplied the estimated effect size (17a). Out of all the research, only 4 (6.1%) reported on the outcomes of additional analyses. Fifty-five publications (83.3%) reported significant negative effects or unforeseen consequences (19).

##### 3.2.1.6 Discussion

Thirty-nine (59.1%) articles reported the limitation of trials (20) and 1 (1.5%) article illustrated the generalizability of the trial findings (21). Just 19 (28.8%) of the research offered an explanation that made sense of the outcome (22).

##### 3.2.1.7 Other information

Twenty-one of the trials (31.8%) provided registration (23) and 36 (54.5%) for sources of funding (25). Only 6 (9.1%) articles, however, included information on how to acquire the entire study protocol (24). The low reporting rate of RCT registration information and protocol information is not conducive for other researchers to fully understand the content of these studies and get detailed references for similar studies.

#### 3.2.2 CONSORT-CHM formulas 2017

Table 2; Figure 3 show the results. The average CONSORT-CHM Formulas 2017 evaluation score is 15.2 among 66 studies, accounting for only 40% of the total 38 items. Compared to CONSORT 2010, CONSORT-CHM Formulas 2017 added one subitem (1c*) and expanded seven items. The results are as follows:

##### 3.2.2.1 Title and abstract

Sixty-six articles (100%) stated by title that the trial targets the specific disease (1a*). The formula’s name, dose form, and TCM pattern were only mentioned in 11 (16.7%) trials (1b*). Only 6 (9.1%) articles determined appropriate keywords, including “Chinese herbal medicine formula” and “RCT” (1c*).

##### 3.2.2.2 Introduction

Statements using TCM approaches were found in sixty-four (97%) papers (2a*), while statements regarding whether the formula takes on a specific disease were found in all 66 (100%) articles (2b*).

##### 3.2.2.3 Methods

Twenty-three research (34.8%) reported on the recruitment of subjects who fit a particular TCM pattern (4a*). Syndrome differentiation and treatment is one of the core features of traditional Chinese medicine. However, the reporting rate of the included RCTs for the TCM pattern was not satisfactory. Even more shocking, none of the articles (0%) described the Chinese herbal medicine formulas in full detail (5*). All RCTs can provide only partial information on Chinese herbal formulas. Twenty-six (39.4%) articles reported the outcome measures related to TCM syndrome in detail (6a*). Other items in CONSORT-CHM Formulas 2017 are consistent with the CONSORT 2010.

##### 3.2.2.4 Discussion

Item 20 has no extension. A discussion of how the formula takes on different TCM patterns on the disease was only performed in 1 (1.5%) article (21*). And 49 (74.2%) studies additionally offered the interpretation with TCM theory (22*).

#### 3.2.3 The checklist of 42 sub-questions based on CONSORT-CHM formulas 2017

According to Table 3; Figure 4, the average score of the checklist of sub-questions based on the CONSORT-CHM Formulas 2017 evaluation is 17.2 among 66 studies, accounting for only 41% of the total 42 items. Only 9 sub-questions had been more than 75% of the included studies fully reported, 13 sub-questions had been more than 50% of the included literature fully reported, and 17 sub-questions had been less than 25% of the literature fully reported. Only 11 (16.7%) studies reported greater than or equal to 50% of the 42 sub-questions. The features found in various sources of CHM formula intervention are as follows:

(1) 5a. For fixed CHM formulas: more than 80% of studies provided information on the CHM formulas’ names, sources, and dosage forms (Q11-Q13). The reported ratio of the dosage of formulas, the treatment duration, and the administration was 66.7%,95.5%, and 100%, respectively (Q25-Q27). Nonetheless, according to Q14–Q17, the percentages for each medicinal substance’s name, source, processing technique, and dosage were 54.5%, 28.8%, 28.8%, and 40.9%, respectively. None of the studies provided the authentication method for each ingredient of CHM formulas (Q18). In twenty-three trials (34.8%), the principles, reasoning, and interpretation of forming the formula(Q19) were mentioned. 56.1%, 57.6%, 33.3%, 3%, and 0% of the studies, respectively, provided references regarding the formulas’ efficacy, the findings of pharmacologic studies, the formula’s manufacturing process, the quality control of both the product and each ingredient in the formula, and the formula’s safety evaluation (Q20-Q24). For the fixed CHM formulas, information on the name of composition, dosage, and course of treatment are highly reported, while the reporting of information about the processing method and dosage of ingredients, processing process of the whole formulas, and safety assessment of formulas is insufficient.
(2) 5b. For individualized CHM formulas: only 9 (13.6%) trials reported the information about how, when, and by whom the CHM formulas were modified (Q28). Other information on formulas is consistent with the results of Q11-Q27.
(3) 5c. For patent proprietary CHM formulas. According to Q29–Q33, the reported ratios for CHM formulas’ composition and dosage, efficacy, safety or quality control, name, the name of the manufacturer, lot number, production date, and expiration date were 18.2%, 39.4%, 4.5%, 33.3%, and 33.3%, in that order. Twenty-eight (42.4%) of the studies stated that the trial’s usage of the patent-protected formula was for a condition that was the same as the reference that was made public (Q34). For patent proprietary CHM formulas, most of them will provide the name of formulas and specific brands, but the specific information of composition, such as name, dosage, and the information of safety, production lot number, and other information, is insufficient.
(4) 5d. Control groups were given a placebo. Just 3%, 3%, and 13.6% of studies discussed how comparable the placebo was to the intervention, how safe and effective the placebo was, and how the placebo was administered in terms of dosage, regimen, and route (Q36-38). None of the trials provided information on the precise manufacturing details of the placebo, including the name and quantity of each ingredient (Q35 and Q39).

### 3.3 Risk of bias

The risks of bias evaluation results for all included trials (Table 4; Figures 5A, B) and two subgroups of RCTs recruiting subjects during and after the first wave of the pandemic are shown in Figures 6A–F. A detailed evaluation of the included studies’ risk of bias can be found in Supplementary Table 7. It was discovered that every RCT that was included had a high or unclear risk of bias in at least one domain. The evaluation of seven bias-prone domains produced the following conclusions: Random sequence generation (selection bias): Because all included studies sufficiently reported random sequence creation, the risk of bias was deemed to be minimal. Allocation concealment (selection bias): Out of the 66 papers that were evaluated, only 8 were deemed to have a low risk of bias, the remaining studies were evaluated with uncertain risk of bias. Fifty-two studies were rated as having an unknown risk of bias, 10 for a high risk of bias, and just 4 for a low risk of bias due to participant and personnel blinding (performance bias). Blinding of outcome assessment (detection bias): Because of inadequate data, the majority of the studies were rated as having an unclear risk of bias, with only 7 for a low risk of bias and 8 for a high risk of bias. Attrition bias, or incomplete result data: 3 studies had a high risk of bias, 4 studies had an unknown risk of bias, and 89% of studies reported complete outcomes and were rated as having a low risk of bias. Selective reporting, often known as reporting bias: just 5% of studies were classified as having a low risk of bias, 17% for a high risk, and 80% for an uncertain risk. Other bias: 9 percent of the trials had a high risk of bias, none had a low risk of bias, and the most had an uncertain risk of bias. In these RCTs, there is a large proportion of unclear risks, especially in the allocation concealment, participant and personnel blinding, blinding of outcome assessment, and selective reporting risks. This may be due to most of the included studies provided incomplete information on study design and methodology.

### 3.4 The difference between RCTs recruited subjects during and after the first wave of the pandemic

Table 4 indicates a statistically significant difference (*P* < 0.05) in ethics approval between the two subgroups of RCTs, which recruited subjects during and after the pandemic’s first wave. Most RCTs recruited subjects after the first wave of the pandemic can provide ethics approval. The three methodological quality assessment checklists and the risk of bias did not significantly differ between these two groups. Our additional research, however, revealed a notable distinction between RCTs published in English that recruited subjects during and after the first wave of the pandemic. For risk of bias, 75% of RCTs published in English that recruited subjects during the first wave of the pandemic had a higher risk of bias in the blinding of participants while only 23% of RCTs published in English recruited subjects after that had a higher risk of bias (*P* < 0.05). Simultaneously, 4 (31%) RCTs in English that recruited subjects after the first wave of the pandemic were identified as low risk of bias in the blinding of participants, but none of the RCTs recruited subjects during the first wave of the pandemic were identified as low risk of bias. These data suggest better reporting of information in clinical registries of RCTs recruited subjects after the first wave of the pandemic than during the first wave of the pandemic. In RCTs published in English, the RCTs that recruited participants after the first wave of the pandemic have higher reporting quality of blinding of participants than those during the first wave of the pandemic.

## 4 Discussion

### 4.1 Main results

The average score of 66 studies of the CONSORT 2010, the CONSORT-CHM Formulas 2017, and the checklist of 42 sub-questions based on the CONSORT-CHM Formulas 2017 evaluation is 16.4, 15.2, and 17.2, respectively. The report rate of sample size calculation, allocation concealment, and blinding is less than 30%. The low reporting rate of CONSORT and CONSORT-CHM formula items and the unclear risk of bias indicates the reporting quality in strictly randomized RCTs on CHM formulas for COVID-19 is inadequate. The checklist of 42 sub-questions based on CONSORT-CHM formulas can be a Supplementary Scale to CONSORT-CHM formulas to help report and assess CHM formula intervention more precisely. The reporting of CHM formula intervention in each medical substance, principles of forming the formula, production method, and safety assessment of formulas are inadequate (all less than 45%). There was a 100% low risk of bias on random sequence generation, and 89% on incomplete result data for all included RCTs. Most studies assessed an “unclear risk of bias” due to insufficient information in the other five domains. In addition, in RCTs published in English, RCTs recruited subjects during the first wave of the pandemic have a higher risk of participant blinding bias than RCTs recruited subjects after the first wave of the pandemic.

### 4.2 Reliability of this study

Because of the ability to reduce or completely eradicate bias, randomized controlled trials (RCTs) are considered the most dependable approach for evaluating therapies. Unreliable treatment effect estimates can result from insufficient reporting and poor design, according to recent methodological evaluations. The CONSORT 2010 statement offers evidence-based, minimal recommendations for standardizing the reporting of RCT results and reducing research bias. It greatly standardizes the publication of RCT results and raises the quality of research papers (Moher et al., 2012). There are more than 600 academic journals around the world that have adopted the CONSORT 2010 statement, which can be used as an important reference for judging whether the article is written in a standardized manner (Chan and Altman, 2005; Plint et al., 2006). Mohamed et al. discovered there was a positive correlation between the level of CONSORT compliance and the impact factor of the studies’ published journals (Jalloh et al., 2024). Based on CONSORT 2010 statement, the CONSORT-CHM Formulas 2017 adds traditional Chinese medicine patterns and items (adds one subitem, and expands the contents of seven items) according to the characteristics of CHM formulas to enhance the clinical RCTs of TCM formulas reporting quality. For studies that have adhered to the CONSORT-CHM Formulas 2017 principle, there is a great improvement in transparency regarding reporting herbal interventions (Ornelas et al., 2018). Another well-known and well-liked method for evaluating the caliber of RCTs in evidence-based medicine is the Cochrane risk of bias tool. In this study, we standardized the scoring criteria of all items through learning and training the CONSORT items scoring to unity of each item judgment criteria. The method of double scoring is adopted. If the two researchers have different judgments, the superior researcher will make the final judgment to ensure accuracy.

### 4.3 The implications of the results of the CONSORT 2010 and the CONSORT-CHM formulas 2017

In this study, we found that a few articles obtained good scores. Four articles obtained a high score of 27 points in CONSORT 2010, and two articles obtained a high score of 24 points in CONSORT-CHM Formulas 2017 evaluation, but the scores of most studies were low. Only 17 articles and 13 articles in CONSORT 2010 and CONSORT-CHM Formulas 2017 reached 20 points respectively, while the proportion of articles with 10 points and below in CONSORT 2010, CONSORT-CHM Formulas 2017 was as high as 3% and 9% respectively. Overall, the methodological reporting quality of the RCTs included needs further improvement.

#### 4.3.1 The analysis from the perspective of CONSORT 2010

Firstly, the title indicates that the corresponding article is an RCT, which can make it more easily identified. However, only 19 articles in this study can be seen as an RCT through the title. Secondly, for most studies, it’s not feasible to study the whole population. Therefore, we need sample size calculation which represents a trade-off between cost effectiveness, ethical concerns, and statistical power (Mohammad et al., 2022). Nevertheless, only 14 (21.2%) articles in this study explain how to calculate the sample size. In this instance, there should be an endeavor to enhance the transparency of sample size calculation to improve the external validity of the RCT. If the sample size calculation report shows little association with the randomized controlled trial, it may be necessary to abandon it, as recommended by Bachetti (Bacchetti, 2002). It is recommended that RCT researchers strictly follow the CONSORT guidelines, use scientific methods to calculate the sample size, and report the calculation of the sample size in detail, including reporting important parameters of the sample size, such as effect size, significance level, statistical power, etc., cite the sample size calculation formula or software used and provide references or software names. Thirdly, relevant studies have demonstrated that blinding is a crucial protective measure to minimize errors (Savovic et al., 2012). Alraddadi et al. indicated that result estimates would be exaggerated if RCTs lack blinding or allocation concealment. In this study, most articles lack a detailed description of the implementation of blinding and allocation concealment mechanisms (Savovic et al., 2012). RCT investigators are advised to strictly follow the CONSORT guidelines, specify the specific method of assigning concealment (such as the use of sealed envelopes or a central randomization system, etc.), identify the independent person responsible for assigning concealment (such as an independent statistician or a third party), and indicate that assigning concealment was performed after subjects were recruited and before the intervention. In addition, report the use of single, double, or triple blindness, explain who was blinded (e.g., subject, investigator, outcome evaluator, etc.), describe the specific operation of the blinding (e.g., placebo or simulated intervention), report whether the blinding was successfully maintained and whether breaking the blinding occurred and why. Authors are encouraged to register experimental protocols in advance and cite them in their articles so that readers are aware of the plan to assign concealment and blind methods. Fourthly, the subjects’ baseline characteristics can be used to reflect the comparability between the experimental and control groups in the results section; 59.1% of the articles in this study elaborated on the baseline characteristics. Fifthly, in the discussion section, only 59.1% of articles elucidated the limitations and extrapolation. The limitations of the study are also an important part of the RCT article. On the one hand, it can demonstrate the objectivity of the research and enhance readers’ trust in the research; on the other hand, the limitations reveal the deficiencies in the study, which can provide suggestions for improvement in future study design, and it points out unresolved problems that can inspire other researchers to carry out relevant studies. Sixthly, the International Committee of Medical Journal Editors (ICMJE) mandates that all clinical trials must be registered to enhance transparency and accountability (DeAngelis et al., 2005). However, only 31.8% of trials in this study were registered. Clinical trial registration can enhance the transparency and credibility of research, promote research ethics and scientific norms, avoid duplication of research and waste of resources, and enhance the credibility and verifiability of research results. In addition, conflicts of interest and the source of financing were stated in 54.5% of the papers. Explaining the source of funding helps readers understand the independence of the research, and disclosing conflicts of interest demonstrates academic integrity. It can also help readers identify bias and interpret the research results more fully. Moreover, only 9.1% of the studies provided a protocol. Protocol improves the transparency and credibility of the research by disclosing the research design and avoiding selective reporting. Moreover, it clarifies the research methods and results, and facilitates other researchers to repeat the experiment or verify the results. Researchers share the experience and lessons of the experiment design, which can promote the progress of the methodology. It is recommended that investigators complete registration with an internationally recognized registration platform (such as ClinicalTrials.gov, WHO ICTRP, China Clinical Trial Registry, etc.) before trial initiation and ensure that registration information is complete, including study purpose, design, sample size, interventions, primary and secondary outcome measures, etc. In the article, report the trial registration number (e.g., NCT number), state the time of trial registration (e.g., before subject recruitment begins), and provide the name and link of the registration platform. It’s recommended that upload the full trial protocol on a registered platform or in an open-access journal (e.g., BMJ Open, Trials), cite the trial protocol in the article, and provide a way to obtain it (such as DOI or link). Therefore, in order to guarantee the protection of subjects’ rights and the validity of the research, we expect that future studies can enhance these areas.

#### 4.3.2 The analysis from the perspective of CONSORT-CHM formulas 2017

CONSORT Formulas 2017 is an expanded version of CONSORT 2010 with some of the same results. The difference is the expanded entry section. To begin with, just 6 publications (9.1%) used “randomized clinical trial” and “traditional Chinese medicine” as keywords. Furthermore, none of the articles provided comprehensive information on the item of “intervention” including the dosage form, source, formula basis, etc., of Chinese herbal medicine formulas. Providing the necessary details and guaranteeing the quality control of CHM is crucial for researchers to evaluate experimental methods and results of CHM-related studies. Chief and deputy botanical drugs are used in conjunction in CHM formulae, which stress the significance of treating and differentiating syndromes. Changes to the drug’s composition, dosage, or manufacturing technique within the same formulation can affect how well the prescription works as a whole. First, reporting detailed information on TCM compounds can give readers a comprehensive understanding of the specific content of the intervention, enhance the transparency of the study, support the replication of the trial, and validate the results of the study. Second, detailed information on interventions is available for clinicians to understand how to properly use interventions to support clinical practice. In addition, a detailed description of the composition, dosage, and preparation method of Chinese herbal medicine can promote the standardization of Chinese herbal medicine research and reduce the differences in results caused by different preparation methods. At the same time, the provision of quality control information (such as the origin of the drug, ingredient testing, etc.) can ensure the consistency and reliability of the intervention. Furthermore, only 16.7% of the publications included information on the name and kind of formula utilized, as well as the TCM Pattern, applied, even though all of them provided a detailed description of the outcome indicators. Lastly, a few articles failed to integrate relevant TCM theories into their discussions. The application of dialectical treatment is a fundamental aspect of TCM theory. Using the Chinese herbal medicine formula that is appropriate for the patient and their TCM pattern is a hallmark and tenet of Traditional Chinese Medicine. Consequently, reporting of TCM Patterns and the analysis of TCM theory are essential for the study of the application of TCM intervention.

### 4.4 The checklist of 42 sub-questions based on CONSORT-CHM formulas 2017

The checklist of 42 sub-questions based on CONSORT-CHM Formulas 2017 can be a supplement scale of CONSORT-CHM to help report and assess CHM formulas intervention more precisely. In our study, all the included studies were scored as zero for the item of “intervention” in CONSORT Formulas 2017. However, we can capture and report more precise information about the CHM interventions by using the sub-questions checklist tool. Two articles obtained a high score of 25 points in the checklist of 42 sub-questions based on CONSORT-CHM Formulas 2017. Unfortunately, the scores of most studies were low. The checklist of sub-questions based on CONSORT-CHM Formulas 2017 score of all articles was only 17.2. There were 95.5%,97%, and 83.3%, respectively, of studies that reported the name, the dosage form, and the source of CHM formulas. However, just 36.4% of studies reported recruiting subjects with specific TCM Patterns according to inclusion and exclusion criteria as well as diagnostic criteria. The formula’s foundational ideas, justification, and interpretation were discussed in 34.8% of the publications. The quality of the final product and each ingredient were revealed in 3% of the studies. The lot number, the manufacturer’s name, the proprietary product name (sometimes known as the brand name), and the patent proprietary CHM formulae were shown in 33.3% of the articles. The following topics were not reported: how each ingredient is authenticated; whether the safe assessment of the formula is conducted; what the name of the placebo is; how the quantity of each ingredient in the placebo; or how the formula affects certain TCM patterns or illnesses. In general, the RCTs included reported the information of the formulas’ name, dosage, and duration of the formulas adequately, while the reporting quality of the processing method, dosage, and quality control of the component of formulas, as well as the production process and adverse reactions of the formulas, was low. The method of preparation of the formulas is not reported in detail, which reduces the transparency of the study and may make the findings difficult to verify or replicate. Failure to report quality control standards may lead to doubts about the consistency and reliability of interventions. Failure to report adverse reactions may result in the safety of an intervention not being assessed. It is recommended to fully report detailed information on TCM compounds, improve reporting transparency, strengthen quality control, report adverse reactions and safety, follow the 42 CONSORT-CHM sub-issue criteria, and ensure that all critical information is reported. During the study design and writing phase, methodological experts or peer reviews can be invited to ensure that the study complies with CONCORT-CHM standards.

### 4.5 Risk of bias

The majority of the included studies only supplied partial details about their methodology and research design. It was shown that every included study had an unknown or considerable risk of bias in at least one domain. No trial was scored as low risk of bias in all domains. Analogous findings were observed in a prior study that included 64 RCTs of COVID-19 therapy with traditional Chinese medicine. The researcher indicated that the overall quality of RCTs investigating traditional Chinese medicine for COVID-19 was substandard and needs to be improved (Zhou et al., 2023a). Terence et al. found (Quinn et al., 2021) that COVID-19 studies published during the first wave of the pandemic had reporting and methodological issues that may compromise the utility of the research and may cause harm. Firstly, the majority of Chinese studies lack adequate information and unusually assess unclear risk of bias. In contrast, English literature tends to be more detailed. Terence et al. also indicated that RCTs suggested lower quality scores in the COVID-19 papers. 1. Random Sequence Generation: Since we limited the need for strict randomization when we included RCTs, the assigned sequences produced this entry were all low-risk. 2. Allocation Concealment: Most RCTs assign hidden details to the report, so most are rated as having an unspecified risk of bias. A few RCTs have managed to disclose specific methods of allocating concealment, such as using sealed envelopes or central random systems. We encourage more RCT researchers to do this to reduce selection bias. 3. Blinding of Participants and Personnel: Again, most of them are unclear about migration risks. About 20% of RCTs are rated as high risk due to the use of the open-label method. Participants and researchers were aware of groupings that could lead to differences in interventions. RCT investigators are advised to report in detail how blinded they are, using either placebo or dummy interventions. 4. Blinding of Outcome Assessment: The results of risk of bias are similar to Blinding of Participants and Personnel. It is recommended that an independent outcome evaluator be used and that blind implementation be reported in detail to avoid measurement bias. 5. Incomplete Outcome Data: This is the ideal result in the migration risk assessment item. Most are low-risk. The missing data is small and the missing reason has nothing to do with the result, so the impact on the result is small. 6. Selective Outcome Reporting: Most of them are unclear bias risks. About 20% were rated high risk due to non-reporting of some of the preset indicators. It is recommended to pre-register the study protocol and outcome indicators in the test platform, provide registration information in the article, and report all preset results to avoid reporting bias. 7. Other Bias: Most of them were unclear risks, and about 10% were rated as high risks because some of the information was significantly unbalanced in baseline characteristics. It is recommended to fully consider potential offset sources in the design phase, and explain other possible offset and control measures in the paper.

### 4.6 The difference between RCTs recruited subjects during and after the first wave of the pandemic

Researchers found (Jung et al., 2021) that COVID-19 clinical studies have a shorter time to publication and have lower methodological reporting quality scores than control studies in the same journal. These studies should be revisited with the emergence of stronger evidence. That’s an interesting proposition. Therefore, we wanted to explore whether the quality of RCT literature would change in the progress of the epidemic. Our study showed that there were no differences between the RCTs published in Chinese during and after the first wave of the epidemic, while the RCTs published in English showed significant differences in the blinding method. The methodological reporting quality of most RCTs published in Chinese is not high, and we now know that their quality has not changed with the progress of the epidemic. In the studies published in English, RCTs recruited subjects during the first wave of the epidemic did not perform well with strict blinding, however, that improved for the RCTs recruited subjects after the first wave of the pandemic. We think there may be several main reasons: on the one hand, the infection was newly discovered during the first wave of the pandemic, and there was limited understanding of the pathogenicity of the new virus. Considering the safety and compatibility of the subjects, they did not use a strict blind method. On the other hand, strict prevention and control measures at that time limited the implementation of blinding. In addition, given the major threat to human health caused by the COVID-19 epidemic at that time and the need for effective drugs, researchers needed to publish the research results as soon as possible, which may have an impact on the implementation of the blind method.

### 4.7 Limitations and future directions

This study has some limitations. The included RCTs were only published in English and Chinese. The data extraction is solely based on the published paper. This approach means that we cannot capture some preliminary tests with good quality in the test method, that are not reported in the final publication. Therefore, when evaluating the trial quality of such studies, it is necessary to review the study protocol and contact the experimenter for more information.

In the future, RCTs should strictly follow the CONSORT 2010, CONSORT-CHM Formulas 2017 and report detailed methodological information, especially sample size calculation, allocation concealment, blind method, and detailed information of TCM formulas. Investigators are encouraged to register clinical trials before trial initiation, make the study protocol public, and report registration information in their articles. Secondly, improve research design. Studies should be based on scientific sample size calculation methods to ensure that the study has sufficient statistical power, and strict allocation concealment and blind measures should be adopted to reduce the risk of bias. Thirdly, fully report detailed information of Chinese herbal formulas, including the source of herbs, processing methods, quality control, adverse reactions, etc. More studies combined with Chinese TCM theories such as syndrome differentiation and treatment to explain the research results and enhance the characteristics of TCM. Fourthly, among the RCTs included, only 2 RCTs were carried out jointly by domestic and foreign researchers. More international cooperation is needed to carry out multi-center, large-sample RCTs to improve research representativeness and extrapolation. Fifthly, the quality of methodological reporting on RCTs for pandemic infectious diseases needs to be improved in the future. Due to the threat to human health caused by the COVID-19 pandemic and the strong need for effective drugs, the publishing of the results of RCTs became urgent, which may have influenced the quality of methodological reporting on RCTs to some extent. Pandemics occur occasionally, but they are not immune to recurrence. In the face of this situation, we still need higher quality RCTs to provide a solid basis for us to make countermeasures.

## 5 Conclusion

The low reporting rate of CONSORT and CONSORT-CHM formula items and unclear risk of bias indicate the methodological reporting quality in strictly randomized RCTs on CHM formulas for COVID-19 is inadequate. The sample size calculation, allocation concealment, and blinding especially need to improve. The checklist of sub-questions based on CONSORT-CHM formulas can be a Supplementary Scale to CONSORT-CHM formulas to help report and assess CHM formula intervention more precisely. The methodological reporting quality of RCTs published in English and enrolled participants during the first wave of the pandemic is worse than the studies that recruited subjects after the first wave of the pandemic. Despite the crisis caused by the pandemic, RCTs on CHM formulas should comply with established methodological and reporting standards.

## Data Availability

The original contributions presented in the study are included in the article/Supplementary Material, further inquiries can be directed to the corresponding author.
