# Supplementary material for: The methodological reporting quality in strictly randomized controlled trials for COVID-19 and precise reporting of Chinese herbal medicine formula intervention
Source: Front Pharmacol. 2025 Mar 31;16:1532290. doi: 10.3389/fphar.2025.1532290 (PMC11994931; doi:10.3389/fphar.2025.1532290)
Supplement: Supplementary file 2 [file Table1.docx]

Supplementary Material

# Contents

Supplementary Table 1. Search strategies for literature review.

Supplementary Table 2. The standards operating procedure (SOP) of the data extraction of sub-questions based on CONSORT-CHM Formula 2017.

Supplementary Table 3. Level of risk of bias.

Supplementary Table 4. Investigators(A and B)’s CONSORT 2010 scores (see Excel file).

Supplementary Table 5. Investigators(A and B)’s CONSORT-CHM Formula 2017 scores (see Excel file).

Supplementary Table 6. Investigators(A and B)’s sub-questions based on CONSORT-CHM Formula 2017 scores (see Excel file).

Supplementary Table 7. Investigators(A and B)’s judgment of risk of bias (see Excel file).

Supplementary Table 8. The excluded 26 studies are due to the lack of rigorous randomization methods (see Excel file).

Supplementary Table 9. The excluded 126 studies with the grounds for exclusion (see Excel file).

Supplementary Table 10. The data extraction of baseline characteristics of included RCTs (see Excel file).

Supplementary Table 11.The information on Chinese herbal formulas and their composition (see Excel file).

Supplementary Table 12.The information on the names of Chinese herbal formulas composition (see Excel file).

**Supplementary Table 1 Search strategies for literature review.**

Taking PubMed as an example, the detailed search strategy is as follows:

| **#** | **Searches** | **Results** |
| --- | --- | --- |
| 1 | "COVID-19"[Mesh] | 260346 |
| 2 | "SARS-CoV-2"[Mesh] | 166974 |
| 3 | coronavirus[Title/Abstract] OR "corona virus"[Title/Abstract] OR coronavirinae[Title/Abstract] OR coronaviridae[Title/Abstract] OR betacoronavirus[Title/Abstract] OR covid19[Title/Abstract] OR "covid 19"[Title/Abstract] OR nCoV[Title/Abstract] OR "CoV 2"[Title/Abstract] OR CoV2[Title/Abstract] OR sarscov2[Title/Abstract] OR 2019nCoV[Title/Abstract] OR "novel CoV"[Title/Abstract] OR "wuhan virus"[Title/Abstract] OR "COVID-19"[Title/Abstract] OR "COVID-2019"[Title/Abstract] OR "SARS-CoV-19"[Title/Abstract] OR "SARS-CoV-2019"[Title/Abstract] OR "SARS-CoV-2"[Title/Abstract] OR "SARS2"[Title/Abstract] OR "2019-nCoV"[Title/Abstract] | 422015 |
| 4 | 1-3/OR | 430883 |
| 5 | "Medicine, Chinese Traditional"[Mesh] OR "Drugs, Chinese Herbal"[Mesh] | 70341 |
| 6 | Traditional Chinese medicine [Title/Abstract] OR Integration of traditional Chinese and Western medicine [Title/Abstract] OR herbal Drugs [Title/Abstract] OR Chinese Drugs, Plant [Title/Abstract] OR Chinese Herbal, Drugs [Title/Abstract] OR Herbal Drugs,Chinese[Title/Abstract] OR Chinese patent medicine[Title/Abstract] OR traditiongal Chinese medicine[Title/Abstract] OR herbal medicine[Title/Abstract] OR Chinese herbal[Title/Abstract] OR Preparation of Chinese medicine[Title/Abstract] | 60733 |
| 7 | 5 OR 6 | 105275 |
| 8 | "Randomized Controlled Trial" [Publication Type] | 612534 |
| 9 | "Randomized Controlled Trials as Topic"[Mesh] | 173076 |
| 10 | "randomly"[Title/Abstract] OR "randomized"[Title/Abstract] "trial"[Title/Abstract] OR "control"[Title/Abstract] OR "randomized controlled trial"[Title/Abstract] OR "controlled clinical trial"[Title/Abstract] OR "placebo"[Title/Abstract] OR "clinical research"[Title/Abstract] OR “clinical observation” [Title/Abstract] OR “clinical study” [Title/Abstract] OR “clinical efficacy” [Title/Abstract] | 3788300 |
| 11 | 8-10/OR | 4098258 |
| 12 | 4 AND 7 AND 11 ("2019/1/1"[Date - Create] : "2024/04/22"[Date - Create]) | 468 |

**Studies included for final analysis**

**(n=60)**

**-Paper not RCT or not real RCT (n=234)**

**-Meeting abstract and so on (n=45)**

**-Editorial Material (n=1)**

**-Communication (n=1)**

**-Letter(n=7)**

**-Commentary (n=1)**

**-News(n=2)**

**-Wrong intervention or inappropriate group(n=38)**

**-Study protocal(n=6)**

**Full-text articles assessed for eligibility**

**(n=422)**

**Supplementary Table 2. The standards operating procedure (SOP) of the data extraction of sub-questions based on CONSORT-CHM Formula 2017.**

| **Section/topic** | **Extension items** | **Questions for assessment** | **Definition of Fully reported (scored as 1), Partially or not reported (scored as 0), and Not applicable (NA)** |
| --- | --- | --- | --- |
| **Title, abstract,**  **and keywords** | Statement of whether the trial targets a TCM Pattern, a Western medicine–deﬁned disease, or a Western medicine–deﬁned disease with a speciﬁc TCM Pattern, if applicable | Q1. Whether it reported that the trial targeted a specific TCM Pattern in “Title”? | “Full reported” is considered if (1) a specific TCM Pattern wa reported in Title when the trial targeted a TCM Pattern or a western medicine–defined disease with a specific TCM Pattern; or (2) “treatment based on syndrome differentiation” were reported in Title when the trial targeted more than one TCM Patterns or a western medicine-defined disease with several TCM patterns. The sub-question was identified from Title and Methods section in full text at the same time. “Partially or not reported” is considered if no TCM Pattern was reported in Title when the trial targeted a/several TCM Pattern(s) in Methods section in full text.  “Not applicable” is considered if the trial targeted a Western medicine-defined disease. |
|  | Illustration of the name and form of the formula used, and the TCM Pattern applied, if applicable | Q2. Whether the name of the CHM formula was reported in “Abstract”? | “Full reported” is considered if the name of the CHM formula was reported in Abstract. Including (1) the name of fixed CHM formulas; (2) “individualized formulas” “hospital preparation” when it refers to individualized CHM formulas; or (3) trade name of patent proprietary CHM formulas. “Partially or not reported” is considered if (1) the name of the CHM formula was Partially or not reported in Abstract, such as “traditional Chinese medicines”, “Chinese herbal medicine formulas”, “integrative Chinese and western medicine”, or the name of treatment (e.g. “self-regulated CHM formula”, “Yiqi Huoxue therapy”, or “wish with traditional chinese medicines”); or (2) there was no Abstract.  “Not applicable” does not apply to this item. |
|  |  | Q3. Whether the dosage form of the CHM formula was reported in “Abstract”? | “Full reported” is considered if the dosage form of the CHM formula was reported in Abstract. Including (1) it reported the specific dosage form of CHM formulas, such as “pill”, “granule”, “tablet”, etc; (2) name of patent proprietary CHM formulas can indicate the dosage forms, such as “Danshen injection”, “Niuhuang Jiedu Tablet”, or Yiqi Yangblood oral liquid”, etc; (3) the dosage form can be judged based on the specified and detailed production process of CHM formulas; or (4) name of CHM formulas include “decoction” “pill”, and it can be judged as decoction or pills based on the production process or administration routes of CHM formulas. “Partially or not reported” is considered if (1) the dosage form of the CHM formula was Partially or not reported in Abstract, and the production process is not specific with summary words “水煎服/打散/研末”; or (2)there was no abstract. |
|  |  | Q4. Whether the TCM pattern was reported in  “Abstract”? | “Full reported” is considered if (1) a specific TCM Pattern was reported in the Abstract when the trial targeted a TCM Pattern or a Western medicine–defined disease with a specific TCM Pattern; or (2) “treatment based on syndrome differentiation” was reported in Abstract when the trial targeted more than one TCM Patterns or a Western medicine-defined disease with several TCM patterns. The sub-question was identified from the Abstract and Methods section in full text at the same time. “Partially or not reported” is considered if no TCM Pattern was reported in the Abstract when the trial targeted a/several TCM Pattern(s) in the Methods section in full text.  “Not applicable” is considered if the trial targeted a Western medicine-defined disease. |
|  | Determination of appropriate keywords, including “Chinese herbal medicine formula” and “randomized controlled trial” | Q5. Whether the “Chinese herbal medicine formula” presented in “Keyword”? | “Full reported” is considered if the Keywords include the “Chinese herbal medicine formula”. “Partially or not reported” is considered if the Keywords did not include “Chinese herbal medicine formula”; or there were no Keywords.  “Not applicable” does not apply to this item. |
|  |  | Q6. Whether “randomized controlled trials” was presented in “Key words”? | “Full reported” is considered if Keywords include “randomized control trials” or “RCT”. “Partially or not reported” is considered if the Keywords did not include “randomized control trials” or “RCT”; or if there were no Keywords.  “Not applicable” does not apply to this item. |
| **Introduction** | Statement with  biomedical science approaches and/or TCM approaches | Q7. Whether the TCM background and explanation of the disease or the TCM Pattern was reported in “Background”? | “Full reported” is considered if the TCM background and explanation of the disease or TCM Pattern were reported in the Background. “Partially or not reported” is considered if no TCM interpretation about the disease or TCM Pattern was reported in the Background.  “Not applicable” does not apply to this item. |
| **Background and objectives** |  | Q8. Whether the biomedical science explanation and/or TCM rationale about the CHM formula were reported in “Background”? | “Full reported” is considered if (1) the biomedical science explanation and/or TCM rationale about the CHM formula were reported in Background; (2) the research foundation or application history of CHM formulas were reported in Background. “Partially or not reported” is considered if no introduction of the CHM formula was reported in the Background.  “Not applicable” does not apply to this item. |
|  | Statement of whether the formula targets a Western medicine–deﬁned disease, a TCM Pattern, or a Western medicine–deﬁned disease with a speciﬁc TCM Pattern | Q9. Whether the objective or hypotheses focused  on the CHM formula in the treatment of a Western medicine-define disease, a TCM Pattern, or a Western medicine-defined disease with a specific TCM Pattern? | “Full reported” is considered if (1) a specific TCM Pattern was reported in objective or hypotheses when the trial targeted a TCM Pattern or a Western medicine–defined disease with a specific TCM Pattern; or (2) “treatment based on syndrome differentiation” was reported in objective or hypotheses when the trial targeted more than one TCM Patterns or a Western medicine-defined disease with several TCM patterns. The sub-question was identified from the objective or hypotheses and Methods section in full text at the same time. “Partially or not reported” is considered if no TCM Pattern was reported in the objective or hypotheses when the trial targeted a/several TCM Pattern(s) in the Methods section in full text.  “Not applicable” is considered if the trial targeted a Western medicine-defined disease. |
| **Methods** | Statement of whether participants with a speciﬁc TCM Pattern were recruited, in terms of 1) diagnostic criteria and 2) inclusion and exclusion criteria. All criteria used should be universally recognized, or reference given to where detailed  explanations can be found. | Q10. Whether the  participants with a specific TCM Pattern were recruited, in terms of 1) diagnostic criteria and 2) inclusion and exclusion criteria, and whether all criteria used were universally recognized, or reference is given to where detailed explanation can be found in “Methods”? | “Full reported” is considered if the participants with a specific TCM Pattern were recruited, in terms of *a.* diagnostic criteria and *b.* inclusion and exclusion criteria. And all criteria used were universally recognized; or reference was given to where a detailed explanation can be found; or a detailed description of the TCM Pattern was provided, such as the symptoms, assessment measures, or scales. “Partially or not reported” is considered if (1)there were no eligibility criteria for participants; (2) there were no eligibility criteria for TCM Pattern, but the trial targeted a TCM Pattern or Western medicine–defined disease with a specific TCM Pattern; or (3) there were no recognized criteria, reference, or detailed description of the eligibility criteria for TCM Pattern in Methods section.  “Not applicable” is considered if the trial targeted a Western medicine-defined disease. |
| **Participants** |  |  |  |
| **Interventions** | 1. Name, source, and dosage form (e.g., decoctions, granules, powders) | Q11. Whether the name of the CHM formula was reported in “Methods”? | “Full reported” is considered if the name of the CHM formula was reported in Method. Every formula should be reported the name if more than one CHM formula was applied in the trial. “Partially or not reported” is considered if (1)it did not report the specific name of the CHM formula, only referred to the “Chinese herbal medicines”, “CHM formula”, or names of TCM therapeutics; or (2) it did not report all names of formulas when several CHM formulas were applied in the trial.  “Not applicable” does not apply to this item. |
| **5a. For ﬁxed CHM formulas** |  | Q12.Whether the source of the CHM formula was reported in “methods”? | “Full reported” is considered if (1) it reported where the CHM formula originated from, such as the classic TCM books, researcher articles, or adapted from a famous classic prescription; (2) it reported who created the CHM formula; or (3) the trial applied a self-made formula along with its detailed explanations for basic formulating principles. If several formulas were applied in the trial, it should report the resources for every formula. “Partially or not reported” is considered if readers can not know about the source of the CHM formula; or it did not report the sources for every formula when several formulas were applied in the trial.  “Not applicable” does not apply to this item. |
|  |  | Q13. Whether the dosage form of the CHM formula  was reported in “methods”? | “Full reported” is considered if the dosage form of the CHM formula was reported in Abstract. Including (1) it reported the specific dosage form of CHM formulas, such as “pill”,“granule”, “tablet”, etc; (2) name of patent proprietary CHM formulas can indicate the dosage forms, such as “Danshen injection”,“Niuhuang Jiedu Tablet”, or Yiqi Youngblood oral liquid”, etc; (3) the dosage form can be judged based on the specified and detailed production process of CHM formulas; or (4)name of CHM formulas include “decoction” “pill”, and it can be judged as decoction or pills based on the production process or administration routes of CHM formulas. “Partially or not reported” is considered if (1) the dosage form of the CHM formula was Partially or not reported in the Abstract, and the production process is not specific with summary words “水煎服/打散/研末”; or (2)there was no abstract. |
|  | 2. Name,source,  processing method, and dosage of each medical substance. names of substances should be presented in at least 2languages: Chinese(Pinyin), Latin,or English. Names of the parts of the substances used should be speciﬁed. | Q14.Whether the name of each medical substance was reported in  “Methods”? | “Full reported” is considered if the name of each medical substance was reported in Methods. “Partially or not reported” is considered if (1)it did not report the name of each medical substance, including there is an “etc.” at the end of the name of the last substance.; (2) it did not report all ingredients of every formula when more than one formula was applied in the trial.  “Not applicable” is considered if only the patent proprietary CHM formula was applied in the trial. |
|  |  | Q15.Whether the source of each medical substance was reported in “Methods”? | “Full reported” is considered if the source of the medical substance was reported in methods. Including (1) At least the sources of  one substance were reported; (2) the sources of some TCM substances are included in their name, such as “Hang Baiju” which means the white Chrysanthemum that is made in Hangzhou, Zhejiang province. “Partially or not reported” is considered if it  did not report the source of the medical substance.  “Not applicable” is considered if only the patent proprietary CHM formula was applied in the trial. |
|  |  | Q16. Whether the  processing method of  each medical substance was reported in “Methods”? | “Full reported” is considered if the processing method of the medical substance was reported in Methods. Including (1) At least the processing methods of one substance were reported; (2) the processing methods of some TCM substances are included in their name, such as “Cu Banxia” which means the Rhizoma Pinelliae that has been prepared with vinegar; (3) it did not report the specific processing methods of substances, but the production criteria was provided, such as “the Rhizoma Pinelliae meet the criterion that was described in *Chinese Pharmacopoeia*”.“Partially or not reported” is considered if it did not report the processing methods of the medical substance.  “Not applicable” is considered if only the patent proprietary CHM formula was applied in the trial. |
|  |  | Q17.Whether the dosage of each medical substance was reported in “Methods”? | “Full reported” is considered if (1) the dosage of each medical substance was reported in Methods, the units can be gram, tael, liter, or specific number; (2) it reported the ratio of the medical substances. “Partially or not reported” is considered if the dosage of each medical substance was Partially or not reported in Methods; or it did not report the dosages of each substance for every formula when several formulas were applied in the trial  “Not applicable” is considered if only the patent proprietary CHM formula was applied in the trial. |
|  | 3. Authentication method of each ingredient and how, when, where, and by whom it was conducted; statement of whether any voucher specimen was retained, and if so, where they were kept and whether they are accessible | Q18.Whether the authentication method of each ingredient was  reported in “Methods”? | “Full reported” is considered if it reported the authentication method of each ingredient and how, when, where, and by whom it was conducted. “Partially or not reported” is considered if it did not report the authentication method of each ingredient.  “Not applicable” is considered if only the patent proprietary CHM formula was applied in the trial. |
|  | 4. Principles, rationale, and interpretation of forming the formula | Q19. Whether the principles, rationale, and interpretation of forming the formula were reported? | “Full reported” is considered if the principles, rationale, and interpretation of forming the formula were reported. “Partially or not reported” is considered if the principles, rationale, and interpretation of forming the formula were Partially or not reported.  “Not applicable” is considered if only the patent proprietary CHM formula was applied in the trial. |
|  | 5. Reference(s) as to the efﬁcacy of the formula, if any | Q20. Whether the reference(s) as to the efficacy of the formula was presented? | “Full reported” is considered if reference(s) as to the efficacy of the formula was presented. “Partially or not reported” is considered if a reference(s) as to the efficacy of the formula was not presented.  “Not applicable” does not apply to this item. |
|  | 6. Pharmacologic study results of the formula, if any | Q21. Whether the pharmacologic study  results of the formula presented? | “Full reported” is considered if pharmacologic study results of the CHM formula were reported. “Partially or not reported” is considered if the pharmacologic study results of the formula were not reported.  “Not applicable” does not apply to this item. |
|  | 7. Production method of  the formula, if any | Q22. Whether the production method of  the formula was reported? | “Full reported” is considered if (1) the production methods of the formula were reported; (2) it reported the formulas were  produced by the hospital, pharmacy, or pharmaceutical company; or (3) the production criteria with reference(s) of the CHM formula were reported in Methods. “Partially or not reported” is considered if the production methods were Partially or not  reported in Methods.  “Not applicable” is considered if only the patent proprietary CHM formula was applied in the trial. |
|  | 8. Quality control of each ingredient and of the product of the formula, if any. This would include any quantitative and/or  Qualitative testing  method(s); when, where, how, and by whom these tests were conducted; whether the original data and samples were kept,  and, if so, whether they are accessible. | Q23. Whether the quality control of each ingredient and of the product of the  formula was conducted? | “Full reported” is considered if the quality control of each ingredient and of the product of the formula was conducted in the trial. This would include any quantitative and/or qualitative testing method(s); when, where, how, and by whom these tests were conducted; whether the original data and samples were kept, and, if so, whether they are accessible. “Partially or not reported” is considered if the quality control of each ingredient and of the  product of the formula was partially or not reported in the trial.  “Not applicable” is considered if only the patent proprietary CHM formula was applied  in the trial. |
|  | 9. Safety assessment of the formula, including tests for heavy metals and toxic elements, pesticide residues, microbial limit, and acute/chronic toxicity,  if any. If yes, it should be stated when, where, how, and by whom these tests were conducted; if the  original data and samples were kept; and, if so, whether they are accessible. | Q24. Whether the safety assessment of the formula was conducted? | “Full reported” is considered if the conduction of safety assessment was reported in the trial. If the safety assessment of the formula was  reported, it should include the tests for heavy metals and toxic elements, pesticide residues, microbial limit, and acute/chronic toxicity. It should be stated when, where, how, and by whom these tests were conducted; if the original data and samples were kept; and, if so, whether they are accessible. “Partially or not reported” is considered if the safety assessment was partially or not reported in the trial.  “Not applicable” is considered if only the patent proprietary CHM formula was applied in the trial. |
|  | 10. Dosage of the formula, and how the dosage was determined | Q25. Whether the dosage of the formula reported? | “Full reported” is considered if (1) the dosage of the formula was reported in Methods.; (2) it reported the dosage of each medical substance of the formulas; (3) it reported the reference(s) on which the dosage was based; or (4) it reported the name of the manufacturer and lot number of the formula if only patent proprietary CHM formulas were applied in the trial.  “Partially or not reported” is considered if the detailed dosage information of the formula was partially (at least the name or dosage of one substance was not reported) or not reported in Methods.  “Not applicable” does not apply to this item. |
|  |  | Q26. Whether the treatment duration of the CHM formulas was reported in “Methods”? | “Full reported” is considered if the treatment duration of the formula was reported in Methods, including (1) the specific time or (2)the number of cycles (menstrual cycle or treatment cycle) “Partially or not reported” is considered if (1) the treatment duration of the formula was not reported; (2) readers can not learn about the treatment duration, such as the description “treat until the pain goes away”; or (3) it did not report the treat duration of each formula when several formulas were applied in the trial.  “Not applicable” does not apply to this item. |
|  | 11. Administration route  (e.g., oral, external) | Q27. Whether the  The administration route of the CHM formula was reported in “Methods”? | “Full reported” is considered if the administration route of the formula was reported in Methods. We do not infer the  administration routes according to the dosage forms or production methods of formulas. “Partially or not reported” is considered if (1) the administration route of the formula was not reported in Methods; or (2) it did not report the treatment duration of each formula when several formulas were applied in the trial.  “Not applicable” does not apply to this item. |
| **5b. For**  **individualized**  **CHM**  **formulas** | 1. See recommendations  5a 1–11  2. Additional information: how, when, and by whom the formula was modiﬁed | Q28. For trials with individualized CHM  formulas, whether it  reported how, when,  and by whom the  CHM formula was  modified in“Methods”? | “Full reported” is considered if it reported how, when, and by whom the CHM formula was modified when the individualized CHM formula was used as the intervention in the trial. If more than one formula was applied in the trial, it should report the specific medical substances of each formula.  “Partially or not reported” is considered if it did not report the modification details of the individualized CHM formula in the trial, including (1) it described “modify the ingredients of formula based on different TCM patterns” without specific methods and medicines for modification; (2) it described “modify the ingredients of formula based on different symptoms” without specific methods and medicines for modification; (3) it described “modify the formulas based on different TCM patterns” without specific ingredients of each formula.  “Not applicable” is considered if (1) no individualized CHM formula was applied in the trial; (2) more than one fixed CHM formula was applied in the trial, such as it applied the fixed CHM formula A before menstruation and the fixed CHM formula B after menstruation. |
| **5c. For patent proprietary CHM**  **formulas** | 1. Reference to publicly available materials, such as pharmacopeia, for the details about the composition, dosage, efﬁcacy, safety, and quality control of the formula | Q29. For trials with patent proprietary CHM formulas, whether the  composition and dosage reported in “Methods”? | “Full reported” is considered if (1) the composition and dosage of the patent proprietary CHM formula were reported; (2) readers can get the information from the instruction manual based on the trial reports; (3) it reported the composition and the ratio of composition; or (4) it reported the patent proprietary CHM formula as the confidential formula that was applied in the trial. “Partially or not reported” is considered if readers do not know about the composition and dosage of the patent proprietary CHM formula in the trial.  “Not applicable” is considered if no patent proprietary CHM formula was applied in the trial. |
|  | 2. Illustration of the details of the formula, namely 1) the proprietary product name (i.e., brand name), 2) name of the manufacturer, 3) the lot number, 4) the production date and expiry date, 5) the name and percentage of added materials, and 6) whether any additional quality control measures  were conducted | Q30. For trials with patent proprietary CHM formulas, whether the efficacy was reported in “Methods”? | “Full reported” is considered if (1) the efficacy of the patent proprietary CHM formula was reported; or (2) it reported the reference(s) as to the efficacy of the patent proprietary CHM formula. “Partially or not reported” is considered if the efficacy of the patent proprietary of the CHM formula was Partially or not reported.  “Not applicable” is considered if no patent proprietary CHM formula was applied in the trial. |
|  |  | Q31. For trials with  patent proprietary CHM formulas, whether the safety or quality control was reported in  “Methods”? | “Full reported” is considered if (1) the safety or quality control of the patent proprietary CHM formula was reported; or (2) readers can get the information from the instruction manual, references, or additional files based on the trial reports; (3) it reported the name of manufacturer and lot number of patent  proprietary CHM formulas so readers can get the information from open materials. “Partially or not reported” is considered if  readers do not know about the safety or quality control of the patent proprietary CHM formula in the trial.  “Not applicable” is considered if no patent proprietary CHM formula was applied in the trial. |
|  |  | Q32. For trials with patent proprietary CHM formulas, whether the  proprietary product  name (i.e., brand name), name of the manufacturer, and lot  number were reported  in “Methods”? | “Full reported” is considered if the proprietary product name (i.e., brand name), name of manufacturer, and lot number were reported in Methods. “Partially or not reported” is considered if the proprietary product name (i.e., brand name),  name of manufacturer, or lot number were partially or not reported.  “Not applicable” is considered if no patent proprietary CHM formula was applied in the trial. |
|  |  | Q33. For trials with patent proprietary CHM formulas, whether the  production date and  expiry date were reported in “Methods”? | “Full reported” is considered if 1) the production date and expiry date of the patent proprietary CHM formula were reported; or 2) readers can get the information from the  instruction manual based on the trial reports. “Partially or not reported” is considered if readers cannot get the production date and expiry date from the trial reports.  “Not applicable” is considered if no patent proprietary CHM formula was applied in the trial. |
|  | 3. Statement of whether the patent proprietary  formula used in the trial is for a condition that is identical to the publicly available reference | Q34. For trials with patent proprietary CHM formulas, whether the patent-proprietary formula used in the trial is for a condition that is  identical to the  publicly available  reference stated? | “Full reported” is considered if (1) it reported the patent proprietary formula used in the trial was for a condition that was identical to the publicly available reference(s) or the  instruction manual; (2) it reported the trial aimed to explore the efficacy of patent proprietary CHM formula in treating a  different disease or TCM pattern; or (3) it reported that the patent proprietary CHM formula targeted the disease or TCM pattern, and it given the references. “Partially or not reported” is considered if it did not report the patent proprietary CHM formula used in the trial was for a condition that was identical to the publicly available reference(s).  “Not applicable” is considered if no patent proprietary CHM formula was applied in the trial. |
| **5d. Contro groups**  **Placebo control** | 1. Name and amount of each ingredient | Q35. For trials with placebo control, whether the name and amount of each ingredient of the placebo were reported in  “Methods”? | “Full reported” is considered if (1) the name and amount of each ingredient of the placebo were reported; or (2) the name and ratio of each ingredient of the placebo were reported “Partially or not reported” is considered if the name or amount of each ingredient of the placebo was partially or not reported in Methods.  “Not applicable” is considered if no placebo control was conducted in the trial. |
|  | 2. Description of the similarity of placebo with the intervention (e.g., color, smell, taste,  appearance, packaging) | Q36. For trials with placebo control, whether the similarity of placebo with the intervention (e.g., color, smell, taste,  appearance, packaging) was reported in  “Methods”? | “Full reported” is considered if (1) the similarity of placebo with the intervention(e.g., color, smell, taste, appearance,  packaging) was reported; (2) it reported the specific information of both placebo and intervention, and readers can learn about the similarity of them. “Partially or not reported” is considered if there is no information about the similarity of  the placebo with the intervention in Methods.  “Not applicable” is considered if no placebo control was conducted in the trial. |
|  | 3. Quality control and  safety assessment, if any | Q37. For trials with  placebo control,  whether the quality  control and safety  assessment of the  placebo was  reported in “Methods”? | “Full reported” is considered if the quality control and safety assessment of the placebo were reported in Methods.  “Partially or not reported” is considered if there was no information about the quality control or safety assessment of the placebo in Methods.  “Not applicable” is considered if no placebo control was conducted in the trial. |
|  | 4. Administration route,  regimen, and dosage | Q38. For trials with  placebo control, whether the administration route,  regimen, and dosage  of the placebo were  reported in “Methods”? | “Full reported” is considered if the administration route, regimen, and dosage of the placebo were all reported in Methods. “Partially or not reported” is considered if the  administration route, regimen, or dosage of the placebo were partially or not reported in Methods.  “Not applicable” is considered if no placebo control was conducted in the trial. |
|  | 5. Production  information: where, when, how, and by whom the placebo was produced | Q39. For trials with placebo control, whether the production information of the placebo was reported, including where, when, how, and by whom the placebo was produced? | “Full reported” is considered if it reported the production information of the placebo, including where, when, how, and by whom the placebo was produced. “Partially or not reported” is considered if the production information of the placebo was partially or not reported in Methods.  “Not applicable” is considered if no placebo control was conducted in the trial. |
| Outcomes | Illustration of outcome  measures with Pattern in detail | Q40. Whether the  outcome measures  included TCM  indicators in  “Outcome”? | “Full reported” is considered if the outcome measures included TCM-related indicators, and it reported the name, measure methods(e.g. the formulas for calculating the incidence  rate of TCM patterns/symptoms, scales to evaluate the TCM patterns/symptoms, or references to provide the methods to measure TCM-related outcomes), and measure point of the TCM indicators (specific date or indicated point). “Partially or not reported” is considered if the name, measure methods, or measure point of the TCM indicators were partially or not  reported in Methods.  “Not applicable” is considered if no TCM-related outcome was measured in the trial. |
| **Discussion** | Discussion of how the formula works on different TCM Patterns or diseases | Q41. Whether any discussion of how the  formula works on  different TCM patterns or diseases was reported in  “Discussion” | “Full reported” is considered if it discussed how and when the CHM formula would work on different TCM Patterns or different diseases in the Discussion. “Partially or not reported” is considered if it did not report how and when the CHM  formula would work on different TCM patterns or different diseases in Discussion.  “Not applicable” does not apply to this item. |
| Generalizability |  |  |  |
| Interpretation | Interpretation with TCM theory | Q42. Whether any interpretation with TCM theory was reported in“Discussion”? | “Full reported” is considered if the results were interpreted and discussed with the TCM theory in the Discussion.  “Not reported” is considered if there were no any discussion about the TCM theory in the Discussion.  “Not applicable” does not apply to this item. |

*TCM: traditional Chinese medicine; CHM: Chinese herbal medicine

**Supplementary Table 3. Level of risk of bias.**

| **Risk items** | **Low risk of bias** | **Unclear risk of bias** | **High risk of bias** |
| --- | --- | --- | --- |
| 1. Sequence  generation | Randommethods used were described (e.g.,table of random numbers, random block, or computer random number generation) | Information about sequence generation was unknown or difficult to judge | A non-random method in the sequence generation process was indicated |
| 2.Allocation  concealment | Participants and the researchers who recruited subjects were not able to predict the allocation(e.g., center assignment, identical containers,  opaque sealed envelopes) | No specified information about the allocation methods (e.g., only reported theratio ofdifferent groups) | Othermethodsunabletobehiddenwereused to allow participants or the researchers who recruited subjects to predict allocations |
| 3.Blinding ofparticipants and personnel | The study described the trial as blinding and mentioned the methods of blinding to make sure that the allocation of interventions in the study was not known to participants and trial  researchers | Blinding and the methods in the study were not mentioned or used blinding methods without  any descriptions, or related outcome was not reported in the studies | Blinding was not used or was incomplete, or the blinding might be broken |
| 4.Blinding of outcome assessment | The study described the trial as blinding and mentioned the methods of blinding to make sure that the allocation of interventions in the study was not known to outcome evaluators | The study described the trial as blinding and mentioned the methods of blinding tomake sure  that the allocation of interventions in the study was not known to outcome evaluators | Blinding was not used or was incomplete, or the blinding might be broken |
| 5.Incomplee  outcome data | No missing data in the study or the missing data did not affect the results, or the analysis treated the missing data in an appropriate way | Information was incomplete, and it was difficult to judge whether the data was complete | The number of participants and causes of absence between groups in the studies was unbalanced or other causes affected the effect size of the intervention |
| 6.Selective outcome reporting | A study proposal or all desired outcomes in published studies | Difficult todetermine whether there was a risk of selective reporting of results due to incomplete information | Important outcomes were not reported in the studies, or methods of measurement and data analysis for unspecified indicators werereported |
| 7. Other bias | No other sources of bias in the studies | Insufficientinformationtodeterminewhetherto cause an important risk of bias or no good justification or evidence that could lead to bias | Potential biases or some other issues related to particular study designs in the studies |
